# Adhesive cationic hyaluronic acid loading with small extracellular vesicles from IL-6 activated mesenchymal stem cells improves repair of corrosive esophageal injury

**DOI:** 10.1016/j.mtbio.2026.103706

**Published:** 2026-09-23

**Authors:** Dan Luo, Shixian Li, Jianhong Sun, Shaolong Zheng, Zhengkui Huang, Na Xiang, Shan Guo, Zhi Yu, Hong Mei, Na Xu, Xiaohui Song, Baoxiang Wang

**Affiliations:** a Department of Gastroenterology, Wuhan Children's Hospital, Tongji Medical College of Huazhong University of Science and Technology, Wuhan, 430015, PR China; b College of Life Sciences and Health, Wuhan University of Science and Technology, Wuhan, 430065, PR China; c State Key Laboratory of Virology and Biosafety, Center for Emerging Infectious Diseases, Wuhan Institute of Virology, Center for Biosafety Mega-Science, Chinese Academy of Sciences, Wuhan, PR China; d Radiographic Center, Wuhan Children's Hospital, Tongji Medical College of Huazhong University of Science & Technology, Wuhan, 430015, PR China; e Department of Obstetrics, Wuhan Children's Hospital, Wuhan Maternal and Child Healthcare Hospital, Tongji Medical College, Huazhong University of Science & Technology, Wuhan, 430015, PR China

**Keywords:** Corrosive esophageal injury, Mesenchymal stem cells, Extracellular vesicles, Exosome, Hyaluronic acid

## Abstract

The treatment of corrosive esophageal injury (CEI) remains challenging due to recurrent courses, frequent complications, and severe tissue damage. Enhancing the therapeutic potential of mesenchymal stem cell-derived small extracellular vesicles (sEVs) through cytokine preconditioning has emerged as a promising strategy. In this study, functionally enhanced sEVs (E-sEVs) were generated by pretreating umbilical cord-derived mesenchymal stem cells with interleukin-6 (IL-6). E-sEVs exhibited superior capabilities in promoting fibroblast migration and suppressing macrophage-mediated inflammation compared to naïve sEVs. To improve local retention and therapeutic efficacy, E-sEVs were integrated into a cationic hyaluronic acid (CHA) hydrogel with strong bioadhesive properties, forming a composite system termed E-sEVs@CHA. In vitro assays demonstrated that E-sEVs@CHA effectively promoted endothelial cell invasion, exhibited antioxidant activity, and modulated macrophage polarization toward an anti-inflammatory phenotype. In a rat model of CEI, E-sEVs@CHA significantly reduced esophageal inflammation, oxidative stress, and fibrotic remodeling, leading to improved tissue repair and reduced stricture formation. This study presents a novel cell-free therapeutic strategy with translational potential for the management of corrosive esophageal injury.

## Graphical abstract

Adhesive cationic hyaluronic acid loading with small extracellular vesicles from IL-6 activated mesenchymal stem cells (E-sEVs@CHA) were prepared. E-sEVs@CHA can improve the repair of corrosive esophageal injury (CEI) through reducing inflammation, alleviating oxidative stress, and remodeling fibrotic regeneration. Created with BioGDP.com.

**Figure:**
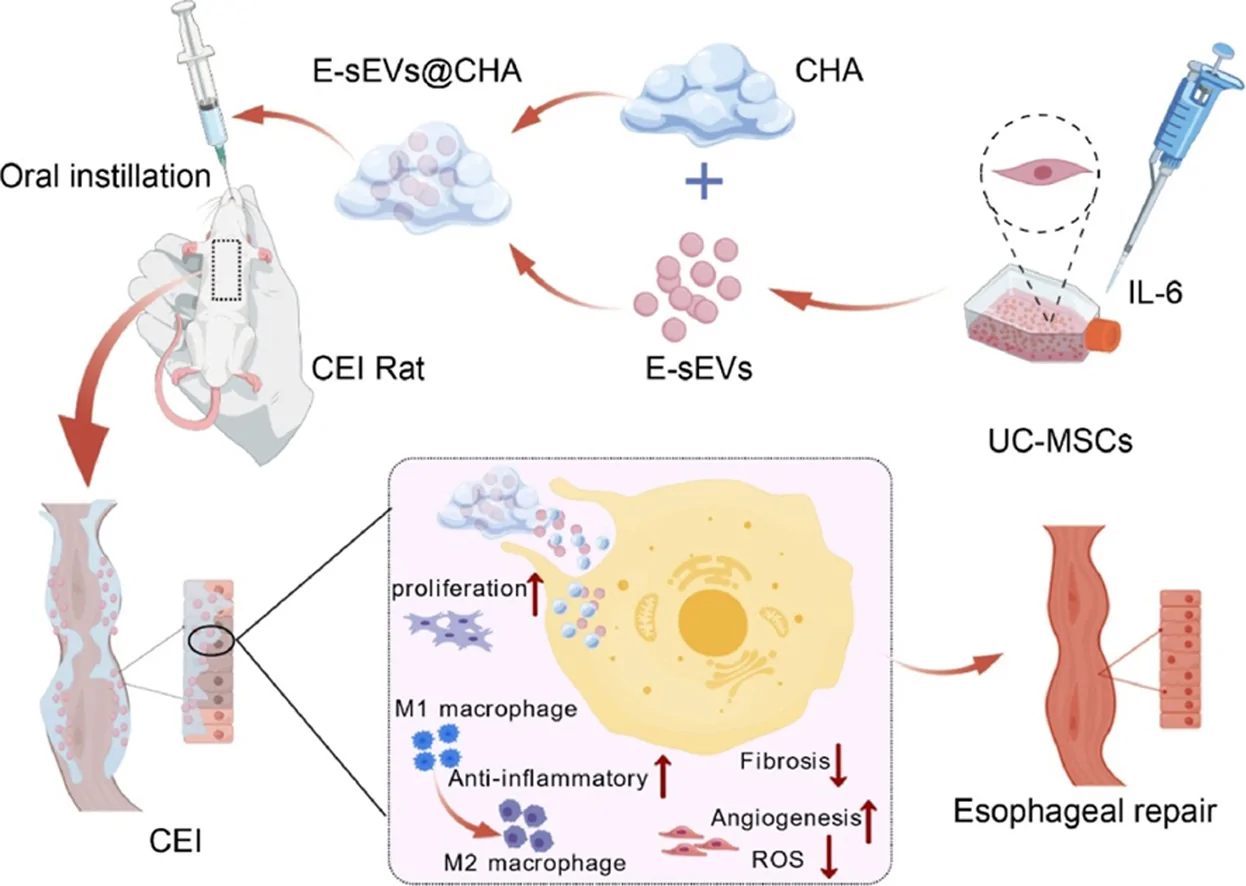

## 1 Introduction

Corrosive esophageal injury (CEI) is a serious injury predominantly resulting from the inadvertent ingestion of corrosive household chemicals, usually acidic and basic substances [1]. CEI carries a high risk of severe complications, with a pooled mortality rate of 6.2% and a stricture formation rate of 24.7% in adult populations, underscoring the serious threat to patients’ lives and long-term quality of life [2]. Current management strategies for CEI include conservative treatment and endoscopic evaluation in the acute phase, followed by endoscopic dilation in the chronic phase, often supplemented by adjunctive intralesional steroid/mitomycin injections or stent placement for refractory strictures, with surgical esophageal reconstruction as the ultimate resort [3]. However, these approaches are hampered by significant drawbacks. Endoscopic dilation frequently requires repeated sessions with limited success and carries a perforation risk ranging from 2.5% to 50%; meanwhile, surgical reconstruction is highly invasive and associated with major complications such as anastomotic leakage and graft redundancy, while pediatric patients face additional long-term challenges including malnutrition, growth impairment, and an increased risk of malignancy [4]. Therefore, it is of great need to advance therapeutic strategies, shifting the treatment paradigm towards earlier, safer, and more effective interventions to promote esophageal wound healing and prevent the development of strictures.

Mesenchymal stem cells (MSCs) have been extensively investigated in regenerative medicine due to their multipotent differentiation capacity and immunomodulatory properties [[5], [6], [7]]. Emerging evidence suggests that the therapeutic benefits of MSCs are largely mediated by their paracrine secretion, particularly small extracellular vesicles (sEVs, also named as exosomes) which contain bioactive molecules including miRNAs, proteins, and lipids [8]. MSC-derived sEVs have demonstrated remarkable capabilities in modulating immune responses [9], promoting tissue repair and angiogenesis [10], and inhibiting fibrotic pathways [11], making them a promising cell-free therapeutic alternative. For instance, studies have shown that MSC-sEVs can promote M2 macrophage polarization, suppress inflammatory cytokine secretion, and reduce collagen deposition in various disease models including systemic sclerosis, myocardial infarction, and liver fibrosis [[12], [13], [14]]. Regarding CEI, direct evidence for MSC or exosome therapy remains limited but emerging. A study by McCloskey et al. demonstrated that submucosal injection of human adipose-derived stem cells at the site of a stented esophageal injury in a rat model significantly enhanced early wound healing, as evidenced by increased granulation tissue formation and more abundant collagen deposition, suggesting that stem cell-based therapy holds emerging potential for improving esophageal repair [15]. Lai et al. demonstrated that adipose-derived MSC exosomes encapsulated in a chitosan/gelatin sponge significantly prevented esophageal stricture formation in a porcine model of esophageal injury, *via* M2b macrophage polarization promoted by exosomal MFGE8 [16]. These findings suggest that MSC or exosome therapy holds promise for preventing esophageal stricture after injury, providing a rationale for exploring its application in CEI.

The therapeutic efficacy of MSCs is increasingly recognized as being dynamically regulated by microenvironmental cues, rather than constituting an intrinsic and invariant property [17]. Upon exposure to an inflammatory milieu, MSCs undergo a functional licensing process, upregulating the expression and secretion of key immunomodulatory factors such as COX-2 and TSG-6, which are critical for suppressing excessive immune responses and promoting tissue repair [18,19]. This phenomenon has been harnessed in vitro through cytokine preconditioning strategies, where replicating an inflammatory environment primes MSCs to enhance their therapeutic potency. Consequently, sEVs derived from these primed MSCs exhibit significantly amplified anti-inflammatory and pro-regenerative properties compared to their naïve counterparts, offering a strategy to overcome the limitations of conventional MSC-sEVs therapy [20,21]. For instance, preconditioning with IFN-γ, TNF-α and IL-1β has been shown to equip MSC-sEVs with superior immunosuppressive effects in models of endometritis or colitis *via* promoting macrophage polarization towards an anti-inflammatory phenotype and increasing regulatory T-cell populations [[22], [23], [24]]. Interleukin-6 (IL-6) is a master regulator of the acute inflammatory response and plays a pivotal role in the early stages of tissue injury [25]. The dysregulation of IL-6 signaling lies at numerous inflammatory disorders, including rheumatoid arthritis [26] or inflammatory bowel disease [27], where it exacerbates inflammation and drives tissue damage. It has been proved that pretreating bone marrow mesenchymal stem cells (BMSCs) with IL-6 significantly enhanced their therapeutic efficacy in ulcerative colitis compared to untreated BMSCs [28]. Therefore, we hypothesize that priming umbilical cord-derived MSCs (UC-MSCs) with IL-6 may generate a uniquely potent population of sEVs (E-sEVs) and this E-sEVs is a promising and logical strategy to develop a more effective, cell-free therapy for improving outcomes in CEI.

To prolong the retention of these E-sEVs on esophagus, it is an effective delivery strategy to use highly adhesive polymers as delivery scaffold. Many kinds of natural and synthetic formulations and hydrogels, such as chitosan, hyaluronic acid, alginate, have been proved to adhere to esophageal tissue, thereby prolonging residence time and enhancing esophageal repair [29,30]. Cationic hyaluronic acid (CHA), a hyaluronic acid derivative modified with positively charged groups, presents an ideal platform. Its inherent positive charge facilitates electrostatic interaction with the negatively charged cell surface and extracellular matrix, thereby enhancing cell adhesion, tissue penetration, and the retention of encapsulated therapeutics at the target site [31,32]. A study showed that CHA-coated spanlastics significantly enhance ocular delivery of cyclosporine A by improving corneal permeation and prolonging retention [32]. Therefore, CHA is also potential to be applied as a drug delivery carrier for treating esophageal injuries.

Herein, in this study, we introduce a novel therapeutic strategy for CEI therapy by combining IL-6-primed MSC-sEVs (E-sEVs) with a cationic hyaluronic acid polymer scaffold (Scheme 1). This integrated scaffold is designed to promote esophageal repair by modulating the local inflammatory response and enhancing tissue regeneration via in-situ released E-sEVs. This combined biomaterial and cell-free therapy holds substantial promise for advancing the treatment of corrosive esophageal injuries, ultimately improving tissue healing and patient prognosis.

**Scheme 1:**
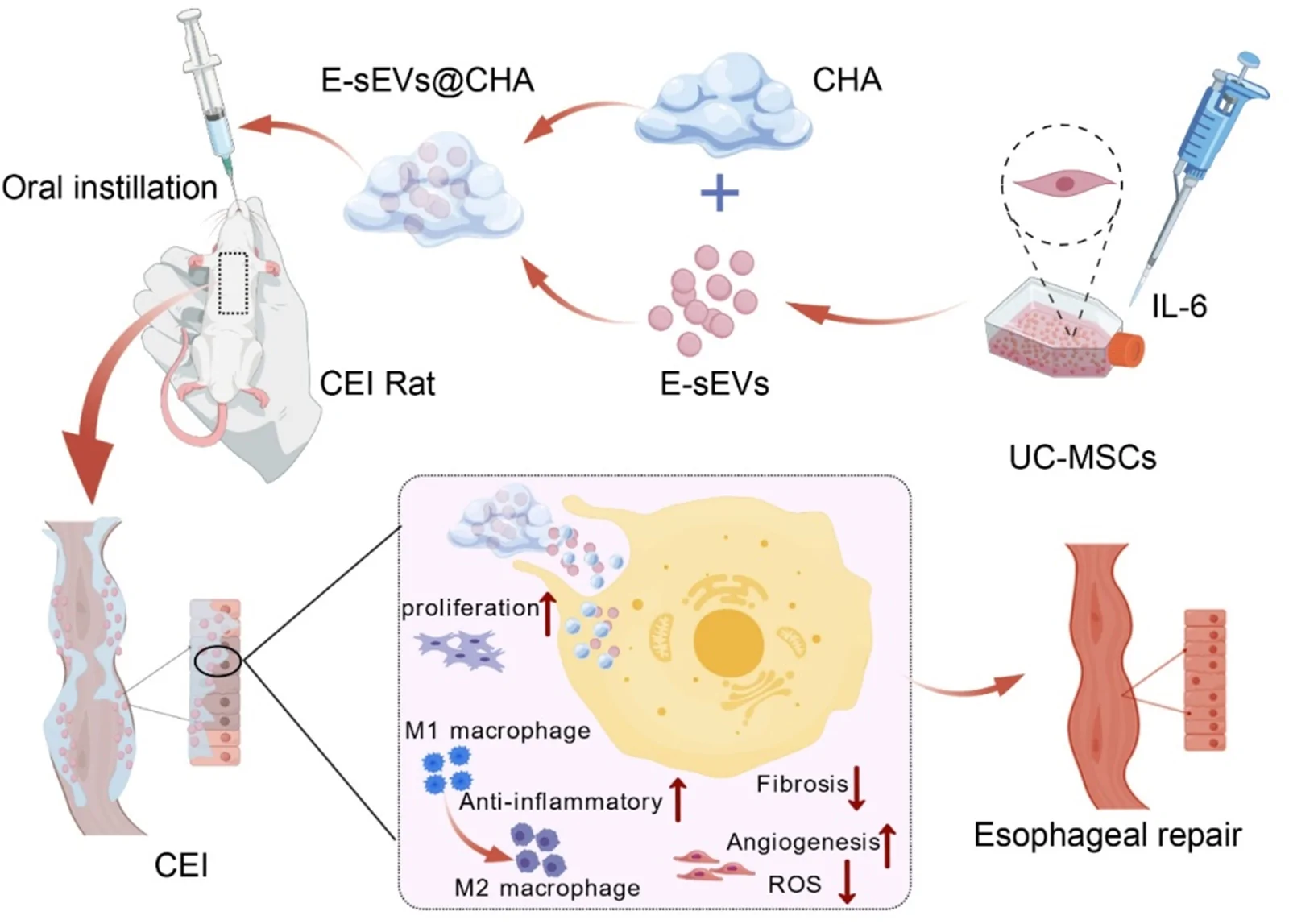
The preparation of E-sEVs@CHA and its therapy in corrosive esophageal injury (CEI). Created with BioGDP.com [31].

## 2 Materials and methods

### 2.1 Preparation and characterization of sEVs and E-sEVs

UC-MSCs (SenBeiJia Biological Technology Co., Ltd) were cultured in α-MEM medium supplemented with 10% fetal bovine serum (FBS), and passages 4-6 were used for the isolation of sEVs and E-sEVs. When cell confluence reached to 70%, cells were stimulated with FBS-supplemented medium containing 10 ng/mL human IL-6 (PeproTech, USA) for 24 h to acquire E-sEVs. After stimulation, serum-free medium was used for additional 48-h culture. The cell supernatant was then collected and subjected to gradient centrifugation at 300*g*, 2000 g, and 10,000 g sequentially. The E-sEVs were subsequently collected from the supernatant by ultracentrifugation at 100,000 g for 90 min. The collected E-sEVs were resuspended in phosphate-buffered saline (PBS) and stored at −80 °C. The sEVs were isolated from the culture supernatant of UC-MSCs without IL-6 stimulation.

The morphology of E-sEVs and sEVs was observed using transmission electron microscopy (TEM, Thermo Fisher Scientific, USA). The particle size distribution, zeta potential, and concentration were determined using a nanoparticle analyzer (RESUN-G03, Resun Tech, China). The total protein quantification analysis was performed using the bicinchoninic acid protein assay (Beyotime, China). The protein content per particle was calculated. The protein markers of sEVs and E-sEVs, CD63 and TSG101, were detected by western blotting method using the antibodies of anti-CD63 (HUABIO, China), anti-CD9 and anti-TSG101 (CST, USA), and β-actin was used as a control.

### 2.2 Cell uptake of sEVs and E-sEVs

sEVs and E-sEVs were stained with PKH67 dye. Human esophageal epithelial cells (HET-1A) were seeded in 24-well plates at a density of 1 × 10^4^ cells/well. After cell attachment, the culture medium was removed, and 500 μL PKH67-labeled sEVs or E-sEVs was added. The cells were then incubated in the dark for 10 h at 37 °C in a cell culture incubator. Subsequently, the cells were stained with Actin-Tracker Red for 30 min, followed by DAPI for 10 min. After each staining step, the cells were washed thoroughly with PBS to remove any unbound dye. Cellular uptake of sEVs was visualized and recorded using an inverted fluorescence microscope.

### 2.3 Preparation and characterization of cationic hyaluronic acid (CHA)

The structure of CHA (Shandong Focusfreda Biotech Co., Ltd, China, cosmetic grade, degree of cationization = 23%) was displayed in Fig. S1. It was added into PBS solution at room temperature and vortexed until completely dissolved. After standing at room temperature for 24 h to completely eliminate bubbles, CHA viscous fluids at various concentrations of 5%, 10%, 15%, and 20% were obtained. The morphology of CHA at different concentrations was observed using a scanning electron microscope (SEM, Thermo Fisher, USA) after freeze drying. The viscosity of CHA was measured using a rotational rheometer (Thermo Scientific, USA) to evaluate the fluid or hydrogel state. To comprehensively assess the broad-spectrum adhesion of CHA, four types of surfaces were selected to represent biological interfaces with distinct wettability and roughness: paper (hydrophilic/rough), plastic plate (hydrophobic/smooth), latex glove (hydrophobic soft tissue), and porcine skin (biological tissue). The CHA samples of different concentrations were placed vertically on these surfaces. The down-sliding behavior of CHA within 30 s was recorded and the sliding distance was measured to evaluate the adhesion ability of CHA for the choice of optimal concentration. To further verify the feasibility of CHA for in vivo animal experiments, a syringe equipped with a gavage needle was used to simulate the intragastric administration procedure. The fluidity and injectability of 15% w/v CHA solution through the gavage needle were evaluated to confirm its suitability for intraesophageal instillation.

The vesicle release rate from CHA hydrogels at different concentrations was determined. CHA hydrogels were prepared at 5%, 10%, 15%, and 20% (w/v), and each was mixed with 20 μg of small extracellular vesicles, followed by centrifugation to deposit the vesicle-loaded CHA. Then, 1 mL PBS was added, and the samples were incubated at 37 °C. Supernatants were collected at 30 min, 1 h, 1.5 h, 2 h, 3 h, 6 h, and 12 h, with an equal volume of fresh PBS immediately replenished to maintain a constant volume. The vesicle release rate was calculated based on protein concentration determined by BCA assay.

### 2.4 Effects of E-sEVs@CHA on cell proliferation, migration, and angiogenesis

**Preparation of CHA extracts.** The CHA, sEVs@CHA, and E-sEVs@CHA extracts for cell experiment was prepared according to ISO 10993-12. Briefly, 15% CHA was immersed into complete medium with a ratio of 1:10 to obtain CHA extract. The 20 μg/mL sEVs or E-sEVs was added into CHA/PBS solution to obtain sEVs@CHA or E-sEVs@CHA. The sEVs@CHA or E-sEVs@CHA extract was obtained by immersing sEVs@CHA or E-sEVs@CHA into complete medium with the same ratio.

**Live/dead cell assay.** The cytocompatibility of E-sEVs@CHA was determined by live/dead cell assay. NIH 3T3 cells were seeded in 24-well plates at a density of 1 × 10^4^ cells/well. After cell attachment, the culture supernatant was replaced by the complete medium containing sEVs, E-sEVs, CHA extract, sEVs@CHA extract, and E-sEVs@CHA extract. After 48 h culture, the cells were stained using Calcein-AM/PI kit (Beyotime, China) and imaged under a microscope to observe the live/dead cell.

**Cell proliferation assay.** The effect of sEVs on cell proliferation was detected by CCK-8 assay. NIH 3T3 cells were seeded in 96-well plates at a density of 4 × 10^3^ cells/well. The complete medium containing sEVs, E-sEVs, CHA extract, sEVs@CHA extract, or E-sEVs@CHA extract was added into wells. After cultured for 24 or 48 h, cell proliferation was evaluated using the CCK-8 assay kit (Meilunbio, China).

**Wound healing assay.** The effect of E-sEVs@CHA on cell migration was evaluated by wound healing assay. Uniform cell-free wounds were created on confluent NIH 3T3 cell monolayers. Then, 2% FBS-supplemented medium containing sEVs, E-sEVs, CHA extract, sEVs@CHA extract, or E-sEVs@CHA extract was added into wells. Cell migration images were photographed under a microscope at 24 and 36 h. The migration rate was assessed by measuring the wound healing width.

**Transwell assay.** The transwell plate with 3.0 μm pore size (Corning Incorporated, USA) was used to evaluate the effect of E-sEVs@CHA on endothelial cell migration ability. The matrigel was placed onto the upper chamber and the complete medium with sEVs, E-sEVs, CHA extract, sEVs@CHA extract, or E-sEVs@CHA extract were added into the lower chamber. EA. hy926 cells were seeded onto the upper chamber and incubated at 37 °C for 24 h. Then, the medium supernatant was removed, and the chamber was gently washed with PBS to remove residual medium. Cells were fixed with 4% paraformaldehyde for 30 min and then washed three times with PBS. The fixed cells were stained with crystal violet for 15 min. The non-invasive cells on the inner surface of the upper chamber were gently removed using a wet cotton swab. Finally, the chamber was placed under a microscope for photography, and the cells migrated to the back of the chamber were observed and counted.

**Tube formation assay.** The effect of E-sEVs@CHA on promoting angiogenesis was evaluated with tube formation assay. The matrigel (Yeasen, China) was placed flat into 24-well plate to mimic extracellular matrix. Then EA. hy926 cells were seed into wells at a density of 5 × 10^4^ cells/well. The culture medium containing sEVs, E-sEVs, CHA extract, sEVs@CHA extract, or E-sEVs@CHA extract was added into wells. After incubation for 6 h, cells were imaged to observe angiogenesis. The tube formation was measured by using ImageJ software.

### 2.5 Effects of E-sEVs@CHA on anti-inflammation, antioxidant, and antifibrotic activity

**Anti-inflammation assay by qRT-PCR.** RAW 264.7 cells (1.0 × 10^6^ cells/well) were seeded in 6-well plates and stimulated with 100 ng/mL lipopolysaccharide (LPS, Sigma-Aldrich, USA) for 6 h. Then, the complete medium containing sEVs, E-sEVs, CHA extract, sEVs@CHA extract, and E-sEVs@CHA extract was added and incubated for 6 h. The nucleic acid were collected for real-time quantitative polymerase chain reaction (qRT-PCR) to detect mRNA expression levels of inflammatory factors TNF-α, iNOS, IL-10, and Arg-1. The primers were listed in Table 1.

**Table 1:** Primers for qRT-PCR.

Species	Gene	Primer sequence (3′- 5’)	Product size (bp)
Mouse	GAPDH	F: GACAACTCACTCAAGATTGTCAG	23
		R: ATGGCATGGACTGTGGTCATGAG	23
Mouse	TNF-α	F: TAGCCCACGTCGTAGCAAAC	20
		R: ACAAGGTACAACCCATCGGC	20
Mouse	IL-10	F: GCTGTCATCGATTTCTCCCCT	20
		R: GCTGTCATCGATTTCTCCCCT	20
Mouse	iNOS	F: AAGCGCAAAACATTTCCTGGG	21
		R: GGGATTCTGGAACATTCTGTGCT	23
Mouse	Arg-1	F: CGTAGACCCTGGGGAACACTAT	22
		R: TCCATCACCTTGCCAATCCC	20
Mouse	α-SMA	F: CAGCCATCTTTCATTGGGATGGAG	24
		R: CCCCCTGACAGGACGTTGT	19
Mouse	Col I	F: CGATGGATTCCCGTTCGAGT	20
		R: GAGGCCTCGGTGGACATTAG	20
Rat	GAPDH	F: ACATTGTTGCCATCAACGAC	20
		R: CTTGCCGTGGGTAGCGTCAT	20
Rat	TNF-α	F: GGAGGGAGAACAGCAACTCC	20
		R: GCCAGTGTATGAGAGGGACG	20
Rat	IL-10	F: GCTGTCATCGATTTCTCCCCT	20
		R: AGACACCTTTGTCTTGGAGCTTAT	23
Rat	IL-6	F: AGCATGCGACCTCTGTTTGA	20
		R: GCCACACGTTTCTTGGCAAT	20
Rat	SOD2	F: GCTTGAATTGCTTGGACGCT	20
		R: GATTACAGCCTCCCCACAGG	20
Rat	CAT	F: ATGGCTATGGCTCACACACC	20
		R: TTGATGCCCTGGTCAGTCTTG	21
Rat	NQO1	F: CTGGCCAATTCAGAGTGGCAT	21
		R: CAGACAGTCTCCAGACGCTT	20
Rat	IL-1β	F: TGCCACCTTTTGACAGTGATG	21
		R: GATGTGCTGCTGCGAGATTT	20
Rat	Col I	F: CTTTGTGGACCTCCGGCTC	19
		R: AGCTGACTTCAGGGATGTCTTC	22
Rat	α-SMA	F: AGACCTTCAATGTCCCTGCC	20
		R: GTCCAGCACAATACCAGTTGT	21
Rat	TGF-β	F: CGGAATACAGGGCTTTCGCT	20
		R: GACGTTTGGGACTGATCCCATT	22

**Macrophage polarization detected by flow cytometry.** To investigate regulatory effect of E-sEVs on macrophage phenotype conversion, RAW 264.7 cells were used for the following experiments. To detect M0-to-M2 polarization, RAW 264.7 cells were seeded in 6-well plates at 1.0 × 10^6^ cells/well. After adherence, sEVs or E-sEVs were added to 20 μg/mL final concentration. Following 24 h co-incubation, cells were collected, stained with anti-CD86 (M1 marker) and anti-CD206 (M2 marker) antibodies, and then detected by flow cytometry. To detect M1/M2 phenotype conversion, attached RAW 264.7 cells were stimulated with 10 ng/mL lipopolysaccharide (LPS) to induce M1 polarization. After 24 h, sEVs or E-sEVs (20 μg/mL final concentration) was added and co-incubated for 24 h. Cells were stained with the above procedure and then detected by flow cytometry.

**ROS detection.** EA. hy926 cells (5.0 × 10^4^ cells/well) were seeded into 24-well plates and stimulated with 1 mM hydrogen peroxide (H2O2) for 24 h. Then, the cell supernatant was replaced with complete medium with sEVs, E-sEVs, CHA extract, sEVs@CHA extract, or E-sEVs@CHA extract and cultured for 24 h. The ROS generation was detected according to the manufacturer's protocol of ROS detection kit (Beyotime, China). Fluorescence images of ROS were captured under a microscope, and fluorescence intensity was quantitatively analyzed using ImageJ software.

**Mitochondrial membrane potential detection.** HET-1A cells were seeded in 6-well plates and subjected to oxidative stress injury induced by 1 mM hydrogen peroxide (H2O2), followed by treatment with sEVs, E-sEVs, CHA, sEVs@CHA, or E-sEVs@CHA for 24 h. Mitochondrial membrane potential (MMP) was detected using JC-1 staining kit according to the manufacturer's instructions. Fluorescence images were captured under a fluorescence microscope, and the ratio of red fluorescence (JC-1 aggregates, representing normal membrane potential) to green fluorescence (JC-1 monomers, representing depolarization) was calculated to evaluate mitochondrial membrane potential changes and depolarization degree.

**Antifibrotic activity detection.** To evaluate the antifibrotic effect of E-sEVs@CHA, NIH 3T3 cells were seeded in 6-well plates at a density of 5.0 × 10^5^ cells/well and cultured in DMEM supplemented with 10% FBS until adherence. Cells were then stimulated with 10 ng/mL transforming growth factor-β (TGF-β) for 24 h to induce fibrosis, followed by treatment with sEVs, E-sEVs, CHA, sEVs@CHA, or E-sEVs@CHA for another 24 h. Total RNA was extracted and reversely transcribed into cDNA. The mRNA expression levels of fibrosis-related markers, α-smooth muscle actin (α-SMA) and type I collagen (Col I), were determined by qRT-PCR to assess fibrosis level.

### 2.6 Therapy effect of E-sEVs@CHA on CEI rat

All experiments involving animals have been reviewed and approved by the Animal Care Committee of the Wuhan Institute of Virology (permit no. WIVA07202105), in accordance with animal ethics guidelines of the Chinese National Health and Medical Research Council (NHMRC). Female SD rats (3-weeks old, 80-100 g) were purchased from ‌Experimental Animal Center in Hubei Center for Disease Control and Prevention‌. Rats were maintained under specific pathogen-free (SPF) conditions in barrier facilities with strict microbial and parasite control, under a 12-h light/dark cycle. Firstly, the biosafety of CHA was evaluated as follows. Six rats were randomly divided into a control group and a CHA experimental group, with three rats in each group. The control group was administered an equal volume of pure water by intraesophageal instillation, while the experimental group received 200 μL of 15% (w/v) CHA daily for 28 consecutive days. On day 28, the blood was collected via orbital punctur and used to measure liver and kidney biochemical indicators including alanine aminotransferase (ALT), aspartate aminotransferase (AST), AST/ALT ratio, creatinine (CR), uric acid (UA), and urea nitrogen (BUN). Tissues from the esophagus, heart, liver, spleen, lung, and kidney were fixed, embedded in paraffin, sectioned, stained with hematoxylin-eosin (HE), and examined under a microscope for histopathological changes.

Then, the CEI rat model was constructed by intraesophageal instillation of NaOH solution. Different concentrations of NaOH, 10%, 15%, and 20%, were used to optimize NaOH concentration. Esophagus H&E-staining was performed after 7, 14, 21 days to observe the esophageal injury. After instillation for 28 days, esophagography and Masson staining were performed to observe the esophageal stenosis and fibrosis. Finally, 15% NaOH solution was chosen to construct the CEI model. The rats were randomly divided into four groups (n = 9): PBS control group (Ctrl), CEI group (Model), sEVs@CHA treatment group (sEVs@CHA), and E-sEVs@CHA treatment group (E-sEVs@CHA). For groups Model, sEVs@CHA, and E-sEVs@CHA, rats were given by intraesophageal instillation with 150 μL NaOH (15% w/v) to establish a CEI model. Drugs of 150 μL sEVs@CHA or E-sEVs@CHA were administered every other day. Body weight and clinical symptoms (such as general activity, hair condition, and milk intake) were monitored daily. Esophagus tissues were collected on day 8 for subsequent H&E staining. Meanwhile, total RNA was extracted from fresh esophagus tissues, and the expression of inflammation-related factors (IL-1β, IL-6, TNF-α, and IL-10) were detected by qRT-PCR. The remaining rats underwent esophagography on day 28 to assess stenosis severity, followed by sacrifice and tissue collection. Masson staining was performed, and mRNA expression of fibrosis-related markers (TGF-β, α-SMA, and Col I) was determined.

To investigate in vivo retention of sEVs and E-sEVs, they were labeled with DIR fluorescent dye, and then complexed with CHA to construct DIR-sEVs@CHA and DIR-E-sEVs@CHA scaffolds. The scaffolds were administered to BALB/C mice via intraesophageal instillation while DIR-labeled sEVs suspension was used as the control group (Ctrl). Mice were sacrificed at 1 h, 2 h, and 3 h after administration, respectively. Esophageal tissues were isolated, and the IVIS® Spectrum Imaging System (PerkinElmer) was utilized to observe the retention of vesicles in esophageal tissues.

### 2.7 Proteomics and miRNA sequencing analysis

To explore the molecular characteristic differences between E-sEVs and sEVs, label-free quantitative proteomics and small RNA sequencing were performed on both types of extracellular vesicle samples. Sample processing, protein and RNA extraction, sequencing, and preliminary data analysis were conducted by Metware Biotechnology Co., Ltd. (Wuhan, China) to ensure standardization and reliability. Differentially expressed molecules were first identified from the omics datasets. For the small RNA sequencing data, significantly differentially expressed miRNAs (p < 0.05) between E-sEVs and sEVs were identified. Target genes of top 3 differentially expressed miRNAs were predicted using miRanda and qtar software, and then analyzed by KEGG and GO enrichment using matascape platform. For the proteomics data, differentially expressed proteins were analyzed and subjected to functional enrichment analysis using the KEGG and GO databases.

### 2.8 Statistical analysis

All experiments were independently performed three times at least. All data were analyzed using GraphPad Prism software and expressed in the form of mean ± standard. Significant differences were evaluated by Student's *t*-test (unpaired and two-tailed), one-way or two-way ANOVA and p < 0.05 was considered statistically significant. All the schematic diagrams were created with BioGDP.com [33].

## 3 Results

### 3.1 Characterization of sEVs and E-sEVs

The purification procedure of sEVs and E-sEVs is illustrated in Fig. 1A. The morphology, size, zeta potential, and protein content were detected to evaluate the differences between sEVs and E-sEVs. TEM images (Fig. 1B) showed that both sEVs and E-sEVs exhibit circular morphology with double-layered membranes, with no significant morphological differences observed. The average hydrodynamic diameters of sEVs and E-sEVs (Fig. 1C) were respectively 69.3 ± 5.56 nm and 71.0 ± 2.94 nm; the zeta potentials of sEVs and E-sEVs (Fig. 1D) were respectively - (11.0 ± 2.27) mV and - (12.7 ± 1.92) mV, indicating no statistically significant differences in particle size and potential distribution between the two groups. Different from size and potential, E-sEVs showed significant difference in particle concentration and protein content with sEVs. The particle concentration of sEVs was 0.91 × 10^11^ particles/mL, while that of E-sEVs was 1.25 × 10^11^ particles/mL (Fig. 1E). BCA assay showed that the protein concentration of sEVs was 1.08 ± 0.06 mg/mL, and that of E-sEVs was 1.40 ± 0.07 mg/mL (Fig. 1F). These results indicated that IL-6 stimulation significantly increased the particle number and protein yield of exosomes derived from UC-MSCs. Western blot analysis results showed that the specific markers CD9, CD63, and TSG101 of extracellular vesicles were positively expressed with β-actin negatively expressed in both sEVs and E-sEVs, while exactly reversed in parental UC-MSCs (Fig. 1G). The results of the vesicle uptake assay (Fig. 1H) demonstrated that both sEVs and E-sEVs could be effectively internalized by esophageal epithelial cells.

**Fig. 1:**
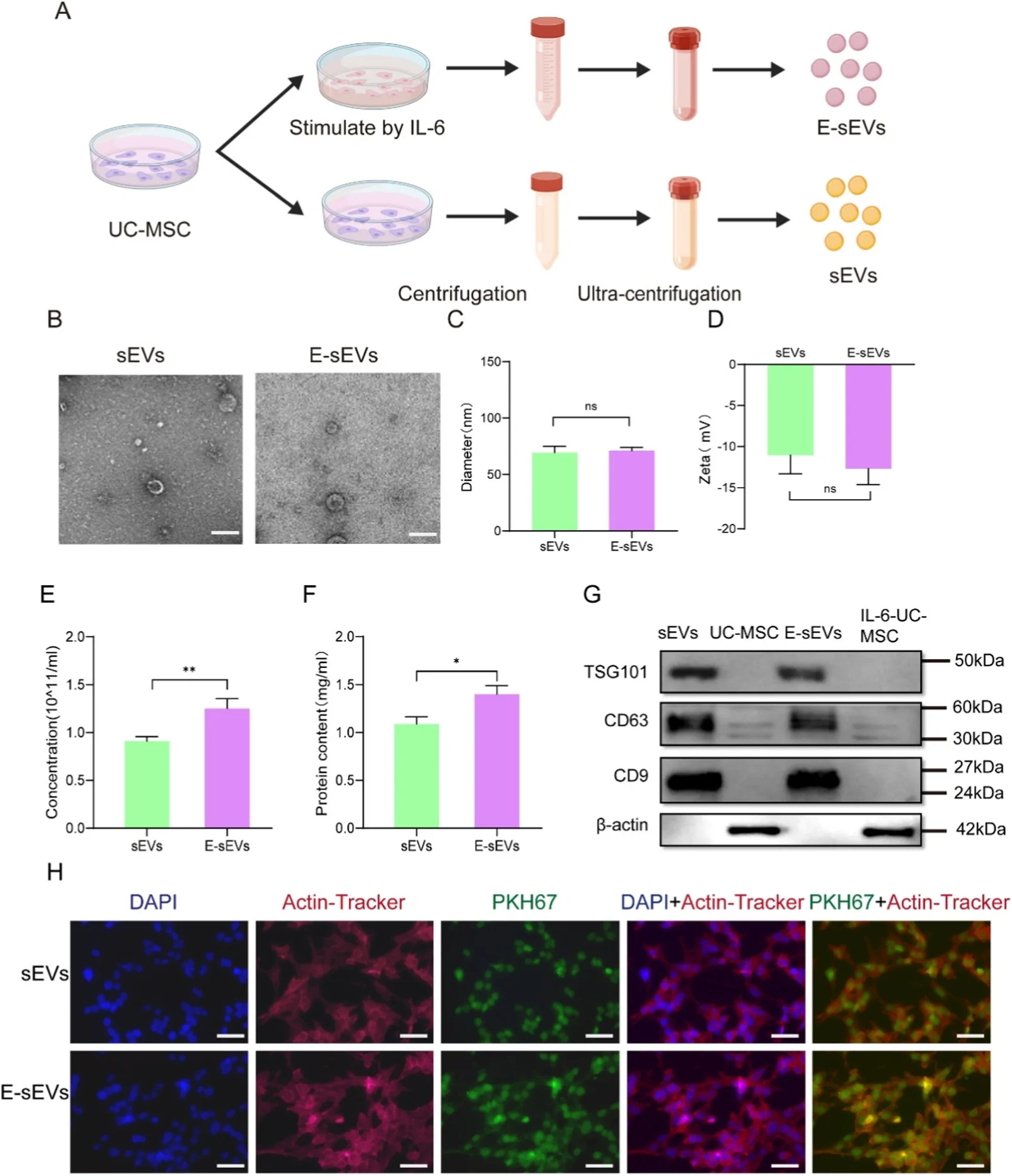
Characterization of sEVs and E-sEVs. (A) Schematic illustration of the preparation procedures. (B) TEM images, scale bars: 100 nm, magnification: ×22,000. (C) Size distributions. (D) Zeta potential. (E) Concentration analysis. (F) Protein content. (G) Western blotting analysis of EV-enriched markers (CD9, CD63, TSG101) and the cellular protein β-actin in UC-MSCs. (H) Cellular uptake of PKH67-labeled sEVs and E-sEVs (green) by human esophageal epithelial cells. Nuclei were stained with DAPI (blue). The cytoskeleton was stained with actin-tracker (Red). n = 3. scale bars: 50 μm (ns means no significance, *p < 0.05, **p < 0.01).

### 3.2 Adhesion and drug-loading abilities of CHA

When employed as a drug delivery carrier for CEI therapy, the essential requirements for CHA are its adhesion and drug-loading capabilities. SEM micrographs of CHA (Fig. 2A) at varying concentrations (5%, 10%, 15%, 20% w/v) demonstrated a direct correlation between concentration and microstructural density, with higher concentrations leading to a more compact internal structure. The rotational rheometer tests (Fig. S2A) showed that the adhesive force of the samples increased with higher CHA concentrations, although the final prepared samples had not yet reached a gel state. The adhesion and flow ability of CHA (Fig. 2B) were visually observed by its natural descent under gravity on four different substrate surfaces (paper, plastic, tin foil, and porcine skin) and the retention length on vertical surfaces within 1 min was measured (Fig. 2C). The retention length decreased as the CHA concentration increased, which means that the drug retention is enhanced with high concentration of CHA. Taking porcine skin as an example, at 5% concentration, CHA flowed rapidly with minimal retention, leading to an insufficient drug residence. In contrast, at a 20% concentration, CHA was nearly immobile, which could easily cause esophagus choking. Therefore, neither of these two concentrations was suitable. It should be noted that the porcine skin may not fully replicate the esophageal mucosal environment, therefore future studies employing ex vivo porcine esophageal tissue or appropriate in vivo models are better to confirm the adhesion properties of CHA under more physiologically relevant conditions. The intraesophageal instillation assay further showed that 15% CHA maintained good injectability (Fig. S2B). After comprehensively considering both adhesion and flow ability, 15% CHA was selected as the delivery scaffold. The E-sEVs@CHA sample was prepared by physically mixing E-sEVs and CHA.

**Fig. 2:**
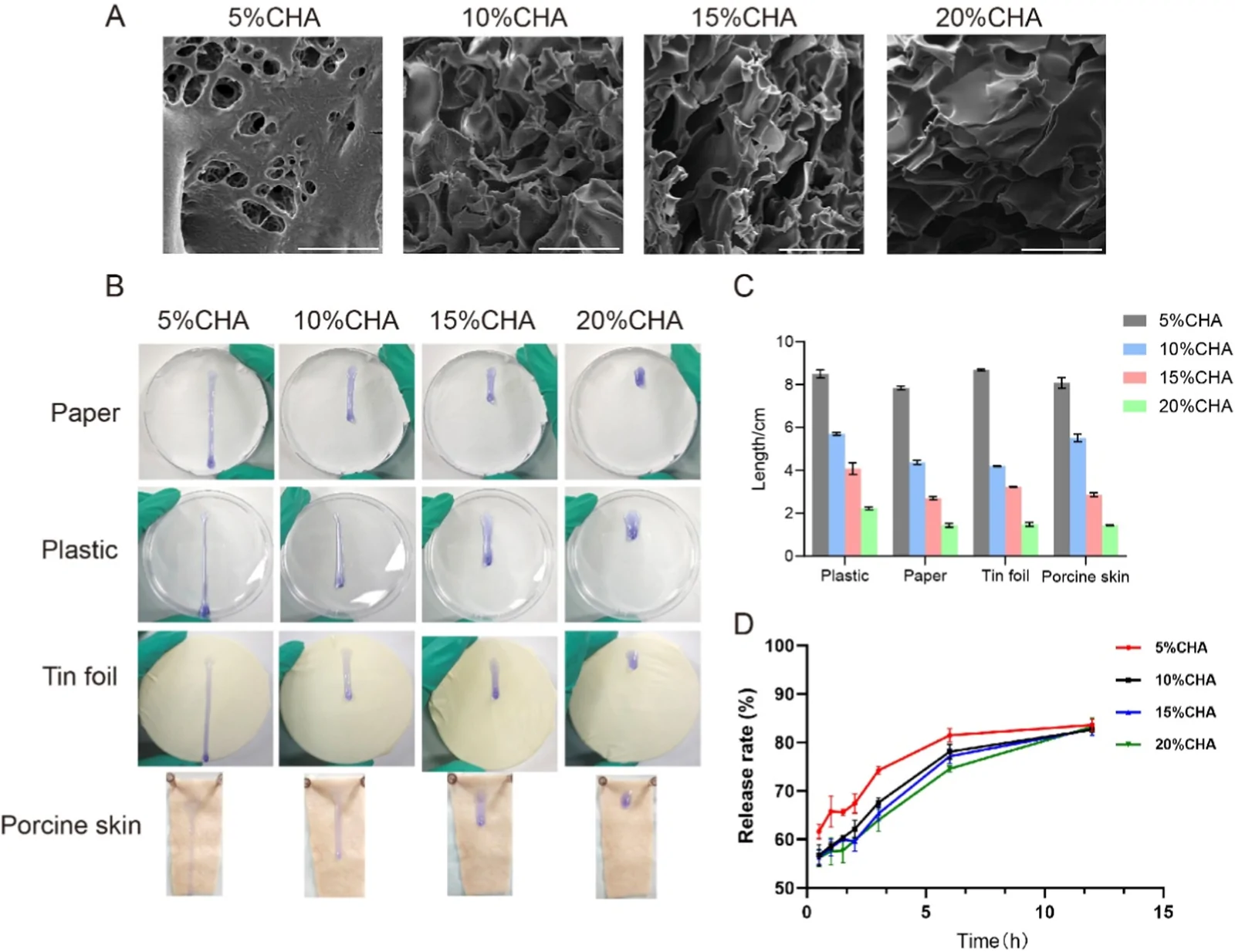
Characterization of CHA at different concentrations (5%, 10%, 15%, 20%; w/v). (A) SEM images, scale bars: 100 μm. (B) Macroscopic images of CHA adhesion on different material surfaces (paper, plastic, tin foil, and porcine skin). (C) Quantitative analysis of retention length of CHA from macroscopic images. (D) Vesicle release kinetics. n = 3.

Vesicle release experiment results (Fig. 2D) showed that all concentrations of CHA achieved a vesicle release rate of more than 50% within 0.5 h, with the 5% concentration group exhibiting the fastest release rate (>60%); notably, the higher the concentration, the slower the release. At 6 h, the release rates of the 10%, 15%, and 20% CHA groups were all higher than 70%, with no statistical differences. The release profile suggested a sustained pattern under simulated conditions, demonstrating the potential of the CHA carrier for in vivo applications.

### 3.3 E-sEVs@CHA promotes cell proliferation, migration, and angiogenesis

The biocompatibility of sEVs, E-sEVs, CHA, sEVs@CHA, and E-sEVs@CHA was evaluated using live/dead cell staining, CCK-8 assay, and hemolysis test. Fluorescence images of AM/PI staining (Fig. S3A) revealed that after co-culturing with NIH3T3 cells for 48 h, nearly all cells in each group were viable (green fluorescence), with no observable dead cells (red fluorescence). Cell proliferation assessed by CCK-8 assay (Fig. S3B) showed that at 24 h, the viability of NIH3T3 cells in the E-sEVs@CHA group exceeded that of the control and CHA groups. After 48 h of culture, cell viability in all treatment groups was higher than that in the untreated control group. Notably, the E-sEVs group exhibited greater cell viability than the sEVs group, and the E-sEVs@CHA group showed the highest viability among all groups. These findings suggest that E-sEVs released from E-sEVs@CHA, in combination with degraded CHA, may synergistically promote fibroblast proliferation. In vitro hemolysis assays (Fig. S3C and S3D) indicated no evident hemolysis in any group, with hemolysis rates all below 2%, which complied with the safety criteria (<5%) for biomaterials. Collectively, these complementary in vitro experiments demonstrate that extracellular vesicles and integrated CHA scaffold possess excellent cytocompatibility with fibroblasts and exhibit no significant cytotoxicity. Furthermore, the results of CHA intraesophageal instillation in rats showed that there were no significant differences in H&E staining of the esophagus heart, liver, spleen, lung, and kidney organs compared to normal rats over a period of 28 days. There were no significant differences in serum liver function indicators ALT, AST, and the ratio of AST/ALT, as well as kidney function indicators CR, UA, and BUN between the control group and the CHA experimental group (Fig. S4). These results provide a solid foundation for subsequent in vivo applications.

The effect of integrated CHA scaffold on cell migration was evaluated by wound-healing assay and transwell assay. The cell scratch images and analysis results (Fig. 3A and D) indicated that after 24 and 36 h of co-culture, the migration rate of NIH3T3 cells followed the same trend: E-sEVs@CHA > sEVs@CHA > CHA > E-sEVs > sEVs > control. This demonstrated that E-sEVs/CHA or sEVs/CHA can synergistically promote cell migration. The transwell assay images and quantitative results (Fig. 3B and E) showed that the trend in EA. hy 926 cell translocation rate was: E-sEVs@CHA ≈ E-sEVs > sEVs@CHA ≈ sEVs > CHA > control. This indicated that the enhanced cell translocation capacity of E-sEVs@CHA and sEVs@CHA is primarily attributable to E-sEVs and sEVs, respectively, with CHA having a minor influence on this specific capability. In addition, the angiogenesis images (Fig. 3C) and quantitative results (Fig. 3F and G) revealed that the number of branches and junctions in the E-sEVs@CHA group was higher than in the sEVs@CHA group, which in turn was higher than in the CHA group. No vascular branches or junctions were observed in the other three non-CHA groups (control, sEVs, and E-sEVs). These findings suggested that CHA alone can induce angiogenesis, and E-sEVs or sEVs in E-sEVs@CHA or sEVs@CHA provided a further synergistic promotion. Collectively, E-sEVs@CHA shows a superior combination for promoting tissue repair.

**Fig. 3:**
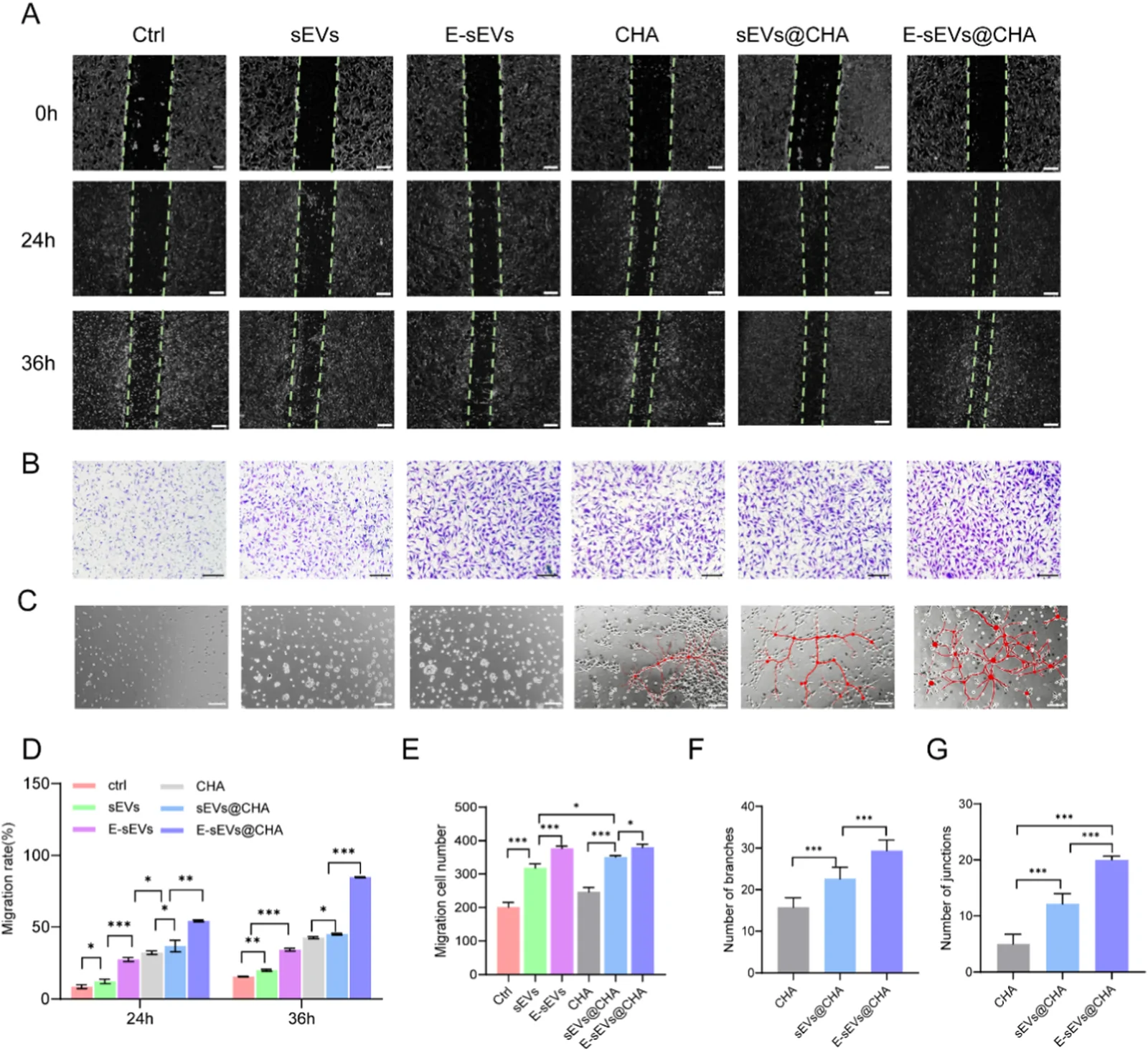
E-sEVs@CHA promotes cell migration and angiogenesis. proliferation and wound repair in NIH3T3 cells, as well as invasion and angiogenesis in EA. hy926 cells. (A) Wound-healing assay images, scale bars: 200 μm. (B) Transwell assay images, scale bars: 200 μm. (C) Angiogenesis assay images with ImageJ Angiogenesis Analyzer plugin, scale bars: 200 μm. Red lines indicate branches and red dots indicate junctions. (D) Quantitative analysis of wound-healing assay (n = 3). (E) Quantitative analysis of transwell assay (n = 3). (F) Quantitative analysis of vascular-like branch number in angiogenesis assay (n = 5). (G) Quantitative analysis of vascular-like junction number in angiogenesis assay (n = 5). (*p < 0.05, **p < 0.01, ***p < 0.001).

### 3.4 E-sEVs@CHA inhibits inflammation and converse macrophage polarization

To investigate the anti-inflammation effect of E-sEVs@CHA, the studies on inflammatory factor expression and macrophage polarization were conducted (Fig. 4A). The results of qRT-PCR showed that the mRNA expression level of the pro-inflammatory factor TNF-α (Fig. 4B) increased upon LPS induction but decreased significantly after treatment in the sample groups. The reduction level in TNF-α expression was greater in the E-sEVs group than in the sEVs group. The expression of the inflammatory marker iNOS (Fig. 4C) increased significantly after LPS treatment but decreased markedly following treatment with the experimental groups. The E-sEVs@CHA and E-sEVs groups exhibited comparable and the lowest levels. On the contrary, the expression level of the anti-inflammatory factor IL-10 (Fig. 4D) was significantly higher in all experimental groups compared to the LPS group, with the E-sEVs@CHA group showing the highest level. The mRNA expression level of the M2 phenotype macrophage marker, Arg-1 (Fig. 4E), showed an early increase upon LPS induction. However, the increase in Arg-1 expression was significantly greater in the experimental groups than in the LPS group alone. The E-sEVs group showed the highest increase, followed by the E-sEVs@CHA group, indicating a potential effect on macrophage phenotype conversion.

**Fig. 4:**
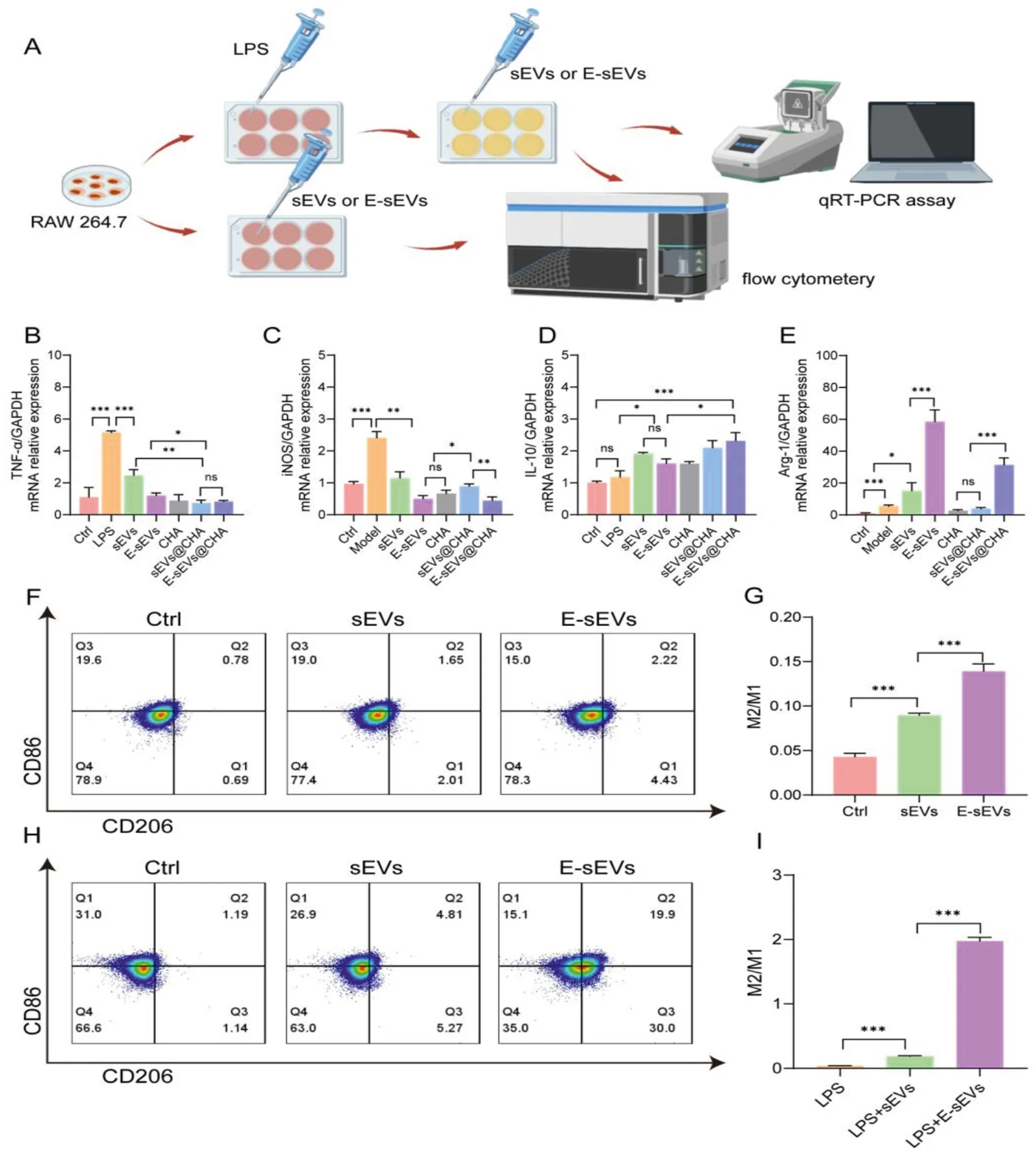
Anti-inflammation effect and regulation on macrophage polarization. (A) Schematic diagram of the experimental procedure. (B-D) qRT-PCR results of inflammatory factors TNF-α, iNOS, IL-10. (E) qRT-PCR result of Arg-1, a M2 phenotype macrophage marker. (F-G) Flow cytometry analysis of macrophage phenotype and quantitative analysis of M2/M1 ratio in macrophages stimulated with E-sEVs@CHA under M0 phenotype; (H-I) Flow cytometry analysis of macrophage phenotype and quantitative analysis of M2/M1 ratio under LPS-induced inflammatory stimulation. n = 3. (ns means no significance, *p < 0.05, **p < 0.01, ***p < 0.001).

Flow cytometry results in M0 macrophage polarization (Fig. 4F and G) demonstrated that E-sEVs significantly elevated the M2/M1 ratio from M0 macrophages (p < 0.001), thus promoting M2 polarization. In the LPS-induced inflammatory model (Fig. 4H and I), E-sEVs markedly conversed M1 phenotype to M2 phenotype and drastically increased the M2/M1 ratio (p < 0.001), with a significantly stronger effect than sEVs. Collectively, E-sEVs effectively drives macrophage polarization toward the M2 anti-inflammatory phenotype under both basal and inflammatory conditions, with a superior effect compared with sEVs. E-sEVs@CHA exhibits favorable anti-inflammatory activity, and E-sEVs alone can effectively regulate macrophage phenotype switching, providing an important basis for regulating inflammation microenvironment in vivo.

### 3.5 E-sEVs@CHA alleviates intracellular oxidative stress

The effect of E-sEVs@CHA on intracellular oxidative stress was evaluated by mitochondrial membrane potential (MMP) and ROS production assays. The MMP (ΔΨm) changes in esophageal epithelial cells induced by H_2_O_2_-mediated oxidative stress were detected JC-1 fluorescent probe (Fig. 5A). The results (Fig. 5B and C) showed that in the control group (Ctrl), JC-1 existed mainly as aggregates with strong red fluorescence and the highest red/green ratio, indicating stable ΔΨm. H_2_O_2_ treatment significantly weakened red fluorescence, enhanced green fluorescence, and sharply reduced the red/green ratio, suggesting obvious ΔΨm depolarization consistent in pathological changes of tissue injury. sEVs, E-sEVs, or CHA alone slightly increased the red/green ratio compared with the H_2_O_2_ group, indicating partial mitigation of mitochondrial damage. In contrast, the sEVs@CHA group showed a further elevated ratio. Notably, the E-sEVs@CHA group had the highest ratio close to Ctrl group, demonstrating superior ability to stabilize ΔΨm compared with single components or sEVs@CHA. Collectively, E-sEVs@CHA effectively alleviates H_2_O_2_-induced damage to esophageal epithelial cells by stabilizing mitochondrial membrane potential.

**Fig. 5:**
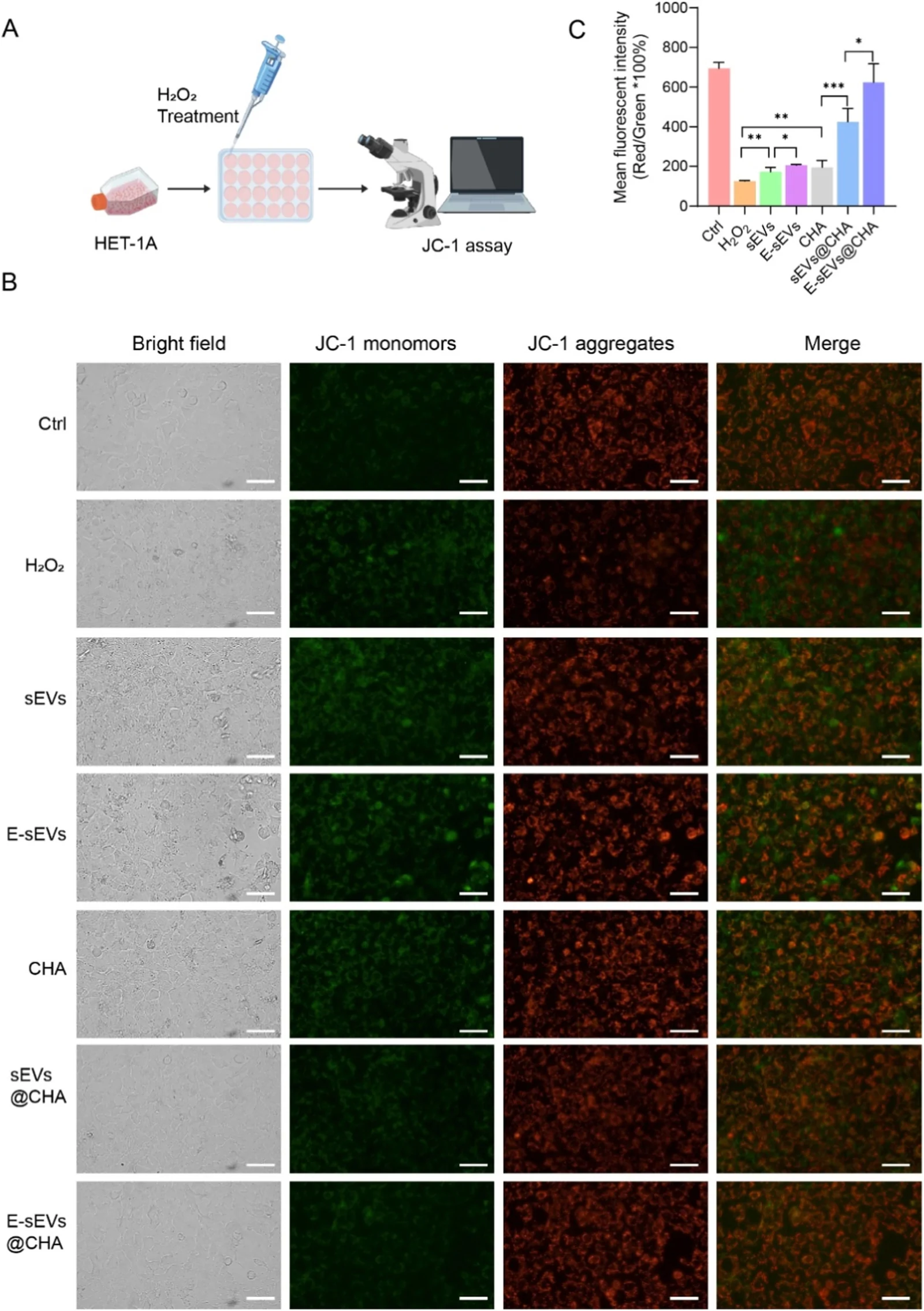
Protective effects on mitochondrial membrane potential (MMP). (A) Schematic diagram of experimental procedure. (B) JC-1 staining images of MMP in HET-1A cells, scale bars: 50 μm. (C) Quantitative analysis results of red-green fluorescence ratio, n = 4. (*p < 0.05, **p < 0.01, ***p < 0.001).

To investigate whether the observed mitochondrial depolarization was associated with oxidative stress, the intracellular ROS levels was further examined (Fig. 6A). The results (Fig. 6B and C) showed that ROS fluorescence intensity was significantly higher in the H_2_O_2_ group than in the control group (p < 0.001), confirming successful establishment of the oxidative damage model. Compared with the H_2_O_2_ group, sEVs, CHA, and E-sEVs all reduced ROS levels; notably, E-sEVs exhibited lower ROS levels than the sEVs and CHA groups. The sEVs@CHA group showed significantly reduced ROS levels, indicating a synergistic antioxidant effect. Importantly, the E-sEVs@CHA group had the lowest ROS levels among all treatment groups (p < 0.01 vs. sEVs@CHA group; p < 0.001 vs. CHA group), demonstrating the strongest antioxidant activity. These results indicated that E-sEVs and CHA component in E-sEVs@CHA can synergistically reduce ROS production, thereby contributing to alleviation of H_2_O_2_-induced intracellular oxidative stress and the promotion potential of tissue repair.

**Fig. 6:**
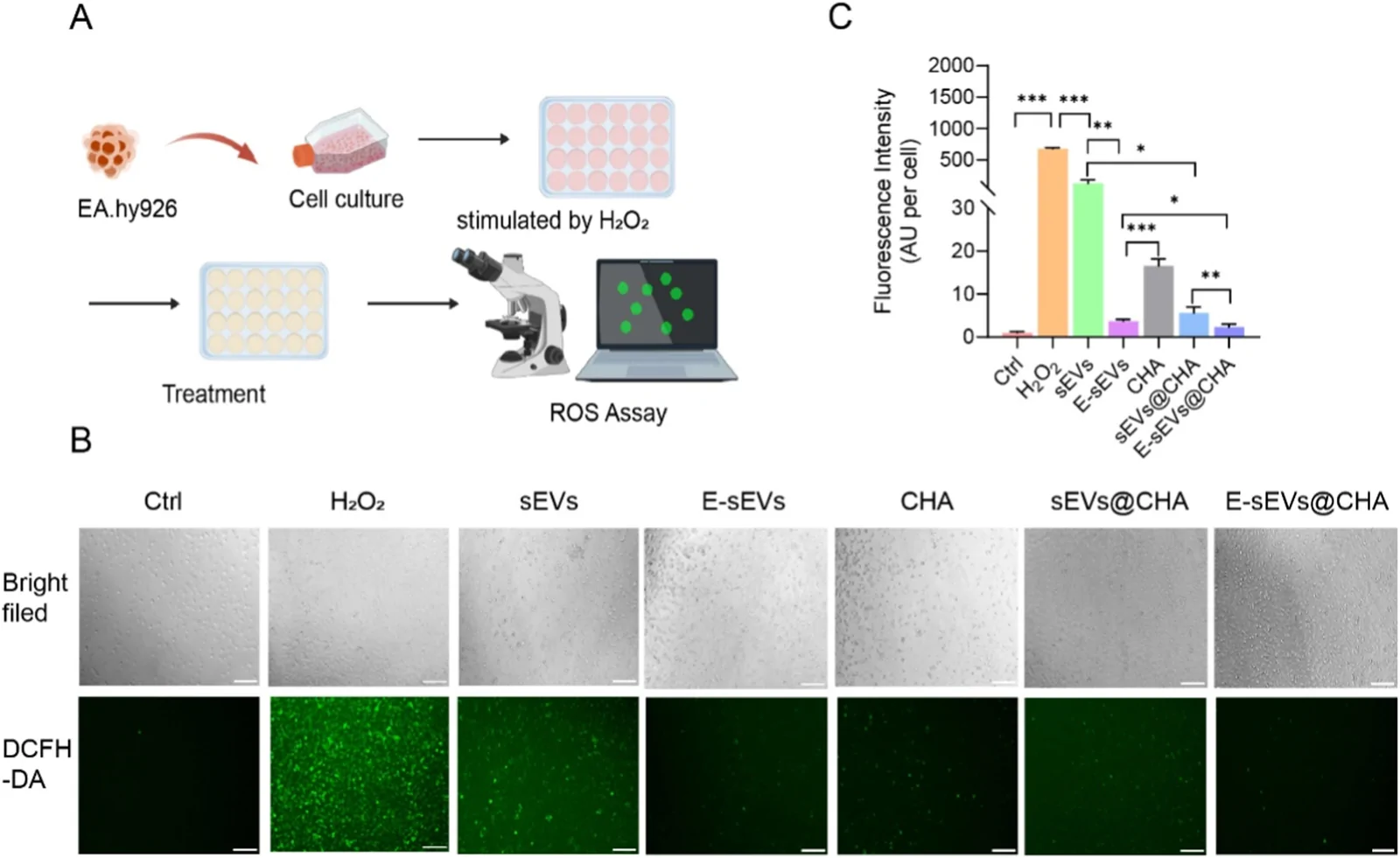
E-sEVs@CHA exerts protective effects on H_2_O_2_-induced oxidative stress in EA. hy926 cells. (A) Schematic diagram of ROS assay procedure. (B) Macroscopic fluorescence images of ROS level, scale bars: 200 μm. (C) Quantitative analysis of ROS fluorescence intensity, n = 4. (*p < 0.05, **p < 0.01, ***p < 0.001).

### 3.6 Anti-fibrotic effect of E-sEVs@CHA

To evaluate the anti-fibrotic effect of E-sEVs@CHA, TGF-β stimulated fibrosis model was performed. The qRT-PCR results (Fig. 7A and B) showed that TGF-β stimulation significantly upregulated the expression of Col I and α-SMA, confirming successful establishment of the fibrotic model. Compared with the TGF-β group, both sEVs and E-sEVs significantly downregulated expressions of Col I and α-SMA, with E-sEVs exerting a stronger inhibitory effect. CHA alone had no significant impact on the expression regulation of Col I and α-SMA. Notably, E-sEVs@CHA treatment further reduced Col I and α-SMA levels, which were significantly superior to those in E-sEVs group. These findings demonstrate that E-sEVs@CHA achieves a synergistic anti-fibrotic effect *via* the downregulation of key fibrotic gene markers, providing a mechanistic basis for its application in post-corrosive fibrosis treatment.

**Fig. 7:**
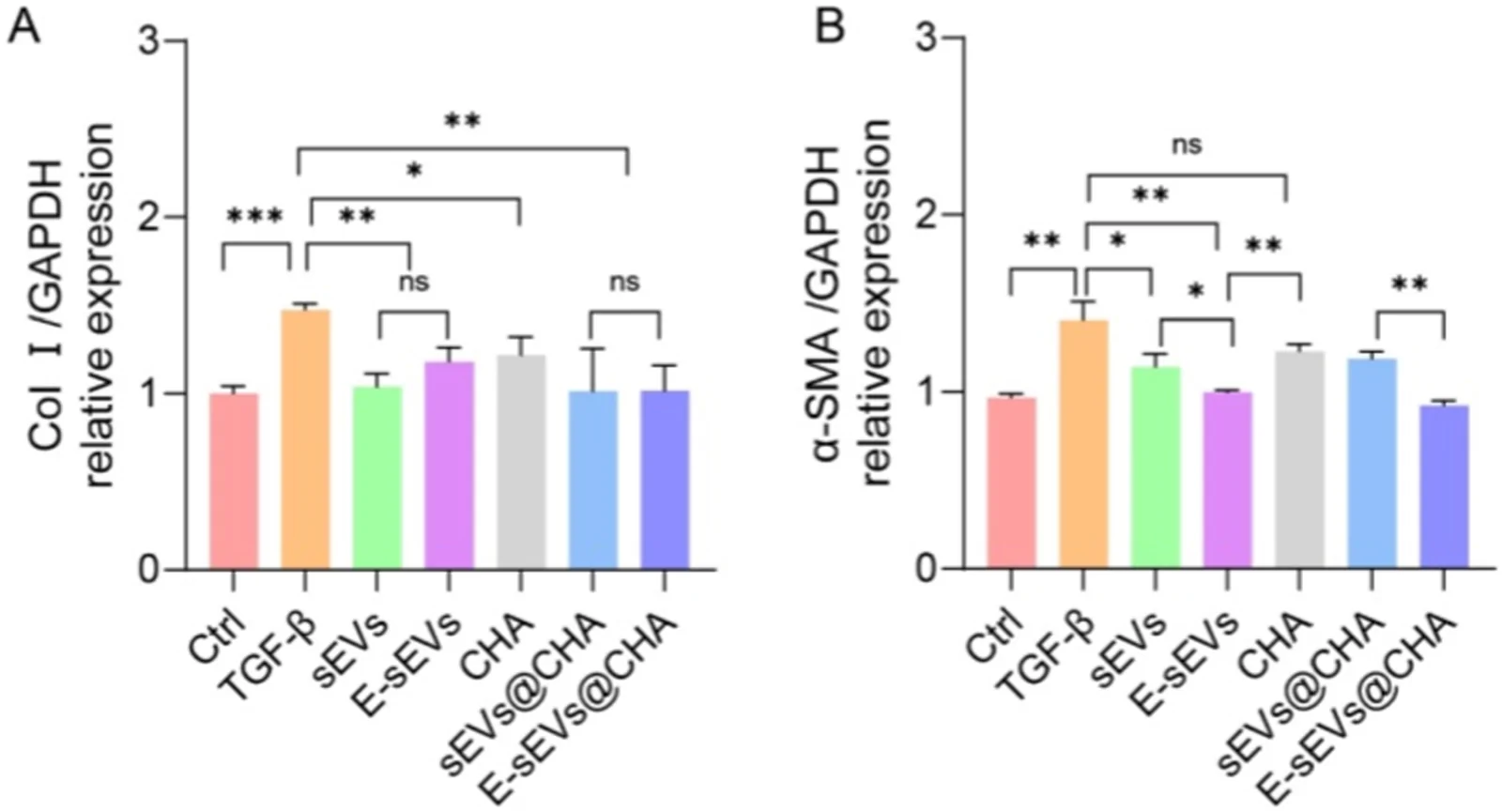
Anti-fibrotic effect detected by qRT-PCR. (A) The mRNA expression level of fibrosis-related factor Col I in TGF-β-induced NIH 3T3 cells. (B) The mRNA expression level of fibrosis-related factor α-SMA in TGF-β-induced NIH 3T3 cells. n = 3. (ns means no significance, *p < 0.05, **p < 0.01, ***p < 0.001).

### 3.7 Therapeutic effect of E-sEVs@CHA on CEI rats

The CEI rat model was established by intraesophageal instillation of NaOH. Three concentrations (10%, 15%, and 20%) were selected to determine the optimal concentration. Esophageal fibrosis and stenosis were used as evaluation endpoint after modeling, detected by H&E-staining, Masson staining and esophagography (Fig. S5). H&E staining (Fig. S5A) showed that 10%, 15%, and 20% (w/v) NaOH all induced esophagus damage to the basal layer to varying degrees, with 10% NaOH causing mild damage and 15% NaOH resulting in obvious injury. Masson staining (Fig. S5B) revealed significant esophageal stenosis, characterized by increased collagen deposition, on day 28 after 15% NaOH injury, which was further confirmed by the visible narrowing in the esophagography images (Fig. S5C, red rectangular frame). Thus, 15% (w/v) NaOH was selected to establish CEI model.

In the therapy experiments, CHA, sEVs@CHA or E-sEVs@CHA treatment was administered every other day after modeling for 28 days (Fig. 8A). Esophagography images (Fig. 8B) revealed the treatment effects on day 28. Severe esophageal stenosis was observed in the Model group, with a significantly narrowed lumen. The CHA group showed limited improvement, while the sEVs@CHA group exhibited slightly milder stenosis than CHA. Notably, the E-sEVs@CHA group, despite residual stenosis, had a significantly widened esophageal lumen and a structure closer to the Ctrl group, indicating superior therapeutic efficacy. Masson staining (Fig. 8C) and quantitative analysis of relative collagen area (Fig. 8D) showed that the Model group had significantly increased collagen deposition compared with the Ctrl group, indicating severe fibrosis. The CHA group showed a slight but statistically insignificant (ns) reduction in collagen area, while the sEVs@CHA group alleviated fibrosis. The E-sEVs@CHA group exhibited a significant reduction in collagen area. Detection of fibrosis-related factors (Fig. 8E) confirmed that the expression of TGF-β, α-SMA, and collagen I was significantly upregulated in the Model group, no improvement with CHA treatment, and partially alleviated in the sEVs@CHA group. Moreover, E-sEVs@CHA significantly downregulated the expression of these factors, thereby highlighting its therapeutic potential in inhibiting fibrosis.

**Fig. 8:**
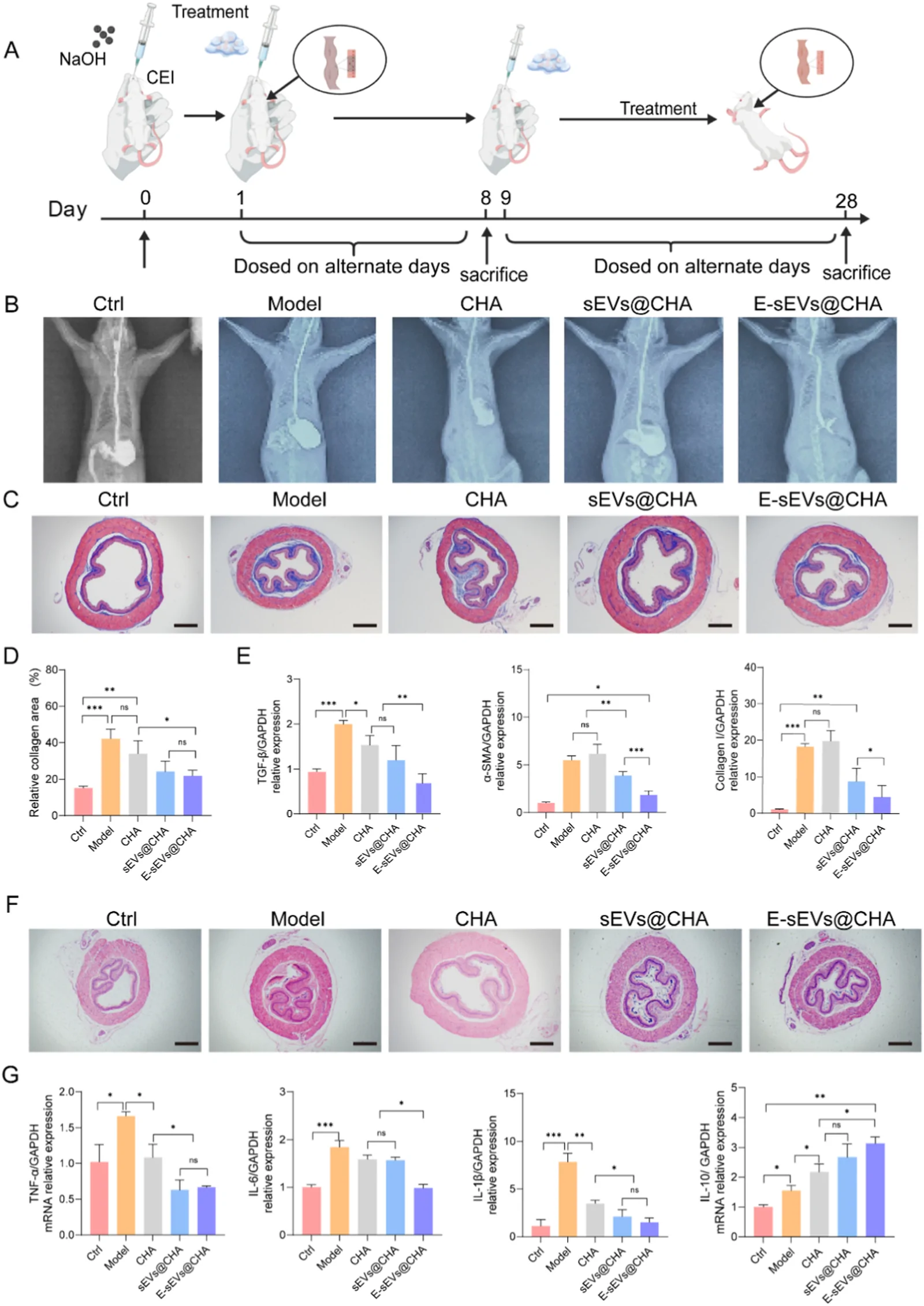
The therapeutic effect in CEI rat model. (A) Schematic diagram of the experimental procedure. (B) Esophagography images on day 28. (C) Masson staining sections on day 28. (D) Quantitative analysis of relative collagen area in Masson staining sections on day 28. (E) The expression levels of fibrosis factors (TGF-β, α-SMA, and Col I) detected by qRT-PCR on day 28. (F) H&E staining sections of esophagus on day 8. (G) The expression levels of inflammatory factors (TNF-α, IL-6, IL-1β, and IL-10) on day 8 detected by qRT-PCR. n = 3. (ns means no significance, *p < 0.05, **p < 0.01, ***p < 0.001).

The H&E staining images of esophagus tissues (Fig. 8F) revealed that the esophageal mucosa and muscularis layers in the model group exhibited structural disruption on day 8, whereas no such structural breaks were observed in either E-sEVs@CHA or sEVs@CHA treatment group. Additionally, the model group showed substantial inflammatory cell infiltration in the esophageal tissue, while the treatment groups displayed relatively milder inflammatory infiltration. The inflammatory levels in esophagus tissues were further validated through RT-PCR experiments. The results (Fig. 8G) showed that the mRNA expression levels of the pro-inflammatory factors IL-1β, IL-6, and TNF-α in the model group were significantly up-regulated compared to the control group. However, after treatment with sEVs@CHA or E-sEVs@CHA, the mRNA levels of these factors were significantly reduced, with E-sEVs@CHA showing a particularly notable inhibitory effect on IL-6 expression. Additionally, the expression level of the anti-inflammatory factor IL-10 in the treatment groups was significantly higher than that in the model group. By suppressing pro-inflammatory cytokines and enhancing anti-inflammatory cytokine expression, E-sEVs@CHA exerted the strongest anti-inflammatory effect, outperforming both the individual components and the sEVs@CHA complex.

The spectrum imaging results (Fig. S6) showed the in vivo retention situation of vesicles. After 1 h of intraesophageal instillation, both the sEVs@CHA and E-sEVs@CHA groups exhibited distinct DIR fluorescent signals in the esophagus, while no fluorescence was detected in the Ctrl group. After 2 h, weak fluorescent signals were observed in the lower segment of the esophagus in the E-sEVs@CHA group. After 3 h, no fluorescence was detected in all three groups. These results indicated that CHA could indeed extend the retention time of extracellular vesicles in the esophagus to a certain extent. All the above results proved that E-sEVs@CHA outperformed all controls by significantly reducing collagen deposition and esophageal stenosis. It demonstrated dual therapeutic benefits, acutely suppressing inflammation and chronically inhibiting fibrosis, positioning it as a promising treatment for esophageal fibrotic diseases.

### 3.8 Molecular mechanism revealed by multi-omics analyses

To investigate the molecular regulatory mechanism of small extracellular vesicles in the repair of CEI, miRNA sequencing on sEVs and E-sEVs were performed with potential repair activity. As shown in Fig. 9A, a total of 168 significantly differentially expressed miRNAs were identified by differential analysis, among which 70 were significantly upregulated and 98 were significantly downregulated in E-sEVs. To explore whether the differential miRNAs play a potential regulatory role in the application of E-sEVs in treating esophageal corrosive injury, top 3 miRNAs, namely hsa-miR-1246, hsa-miR-193a-5p, and hsa-miR-1290, were selected for further discussion. GO functional enrichment analysis (Fig. 9B) showed that these target genes can influence cell behavior by participating in various biological processes, such as the response of cells to glucocorticoids and corticosteroids. They can also regulate cellular components such as the cell body, presynaptic and postsynaptic membranes, neuronal cell bodies, and synapses between neurons, and affect molecular functions such as kinase activity, protein serine/threonine phosphorylation, glycosyltransferase activity, and actin binding. KEGG pathway enrichment analysis (Fig. 9C) further demonstrated that the target genes were significantly enriched in MAPK, PI3K-Akt, cAMP, HIF-1, cellular adhesion molecules, which are all closely related to cell proliferation, migration, inflammation regulation, and tissue repair processes.

**Fig. 9:**
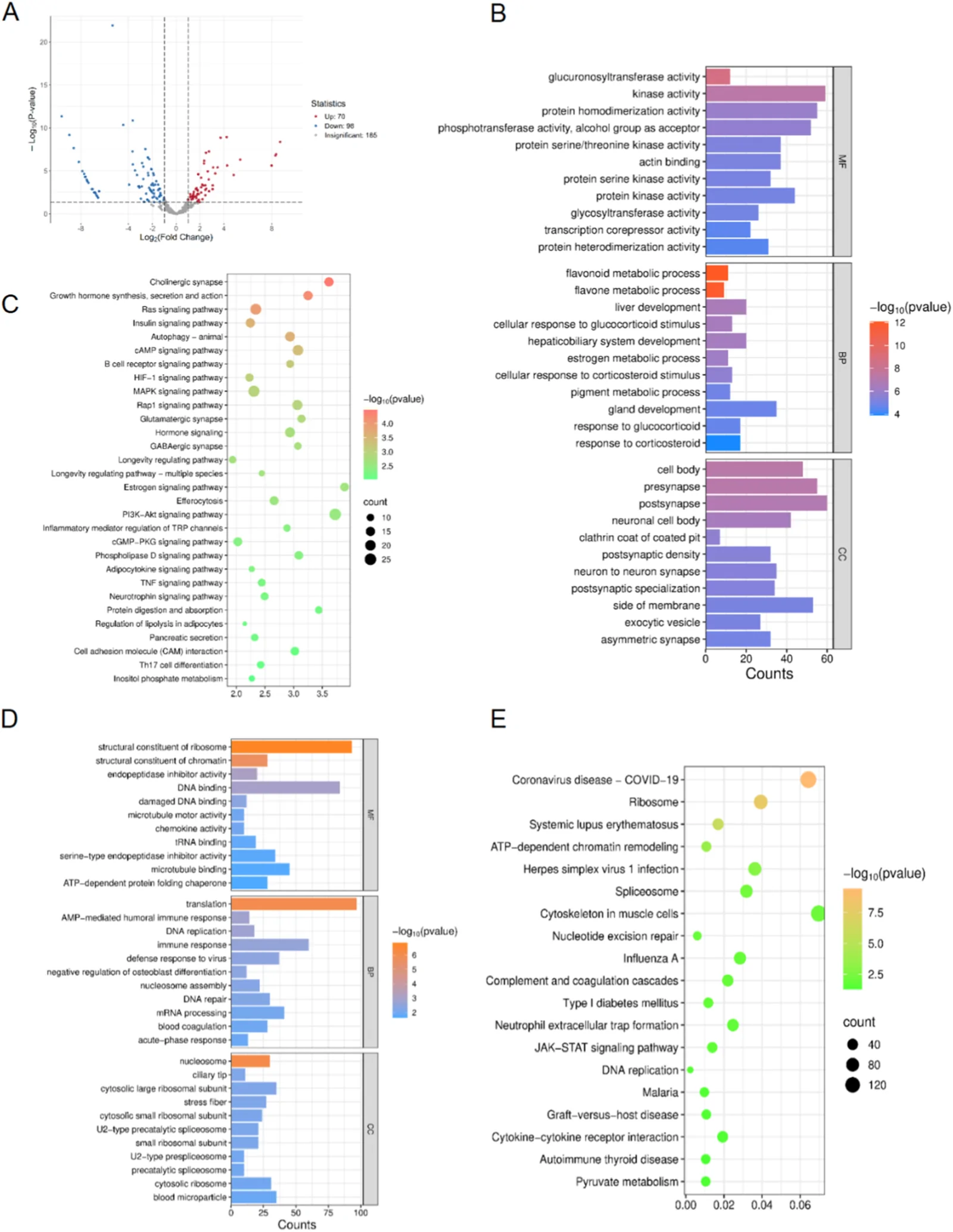
MiRNA sequencing and proteomic analysis of sEVs and E-sEVs. (A) Differentially expressed miRNAs between sEVs and E-sEVs. Red and blue dots represent significantly up- and down-regulated miRNAs, respectively. (B) GO enrichment analysis for target genes of differentially expressed miRNAs. (C) KEGG pathway enrichment analysis for target genes of differentially expressed miRNAs. (D) GO enrichment analysis for differentially expressed proteins between sEVs and E-sEVs. (E) KEGG pathway enrichment analysis for differentially expressed proteins.

For proteomic analysis, significantly different proteins were screened. The results showed that the expressions of proteins such as COL1A2, COL5A1, VCAN, CHI3L1, H2AX, MACROH2A1, CGAS, and the ribosomal and histone families were all showing an upward trend in E-sEVs; while the proteins such as IL6, CCL2, CXCL1, C5, C7, C9, F9, F10, SPP1, LBP, and MX1 showed a downward trend compared to sEVs. The GO functional enrichment analysis (Fig. 9D) indicates that in terms of molecular functions, the core is enriched in ribosome structure composition (structural constituent of ribosome) and DNA binding, and is also involved in activities such as chromatin structure maintenance, endopeptidase inhibition, and microtubule binding; at the biological process level, it is most significantly enriched in protein translation, and is widely involved in key life processes such as immune response, antiviral defense, DNA replication and repair, mRNA processing, and nucleosome assembly; at the cellular component level, it is mainly located in nucleosomes and cytosolic ribosomal large subunits. The KEGG pathway analysis of differential proteins (Fig. 9E) revealed that the differential proteins between E-sEVs and sEVs were significantly enriched in the JAK-STAT signaling pathway. And as the core signal integration hub, the JAK-STAT signaling pathway has extensive cross-talk with the PI3K-Akt, MAPK, and HIF-1 pathways.

## 4 Discussions

Corrosive esophageal injury (CEI) remains a formidable clinical challenge due to its high morbidity, substantial mortality, and the near-inevitable development of esophageal strictures that profoundly compromise patients’ quality of life [2,4]. Conventional interventions, including endoscopic dilation and surgical reconstruction, are frequently limited by inadequate efficacy, considerable invasiveness, and failure to fundamentally address the underlying pathological processes of tissue destruction and fibrotic remodeling [3,4]. Mesenchymal stem cells (MSCs) and their derived small extracellular vesicles (sEVs) have emerged as promising cell-free therapeutic candidates, owing to their inherent immunomodulatory and pro-regenerative properties [[7], [8], [9]]. However, the clinical translation of MSC-sEV therapy faces two critical bottlenecks: (i) the suboptimal therapeutic potency of naïve sEVs, and (ii) the rapid clearance of locally administered therapeutics from the dynamic esophageal environment. Herein, in this study, we developed a composite hydrogel system (E-sEVs@CHA) that strategically addresses these limitations through two integrated innovations: (i) functional enhancement of sEVs via IL-6 preconditioning of parental MSCs, and (ii) targeted retention using a bioadhesive cationic hyaluronic acid (CHA) scaffold. Our systematic investigation, combining comprehensive in vitro mechanistic assays with an in vivo rat CEI model, demonstrates that E-sEVs@CHA exerts multi-stage therapeutic effects, acutely suppressing inflammation and oxidative stress while chronically inhibiting fibrotic remodeling, thereby establishing a translatable strategy for CEI management.

Firstly, the role of IL-6 in the context of MSC biology is complex and context-dependent. IL-6 is not merely a pro-inflammatory mediator [34,35], but also a pleiotropic cytokine that plays essential roles in MSC homeostasis [[25], [26], [27]]. IL-6 preconditioning confers enhanced therapeutic potency to MSC-sEVs. The therapeutic functionality of MSCs is increasingly recognized as dynamically regulated by microenvironmental cues, rather than constituting an intrinsic invariant property [17]. Harnessing this phenomenon, we employed IL-6, a master regulator of acute inflammatory responses [25], to precondition umbilical cord-derived MSCs, generating functionally enhanced E-sEVs. Our results demonstrate that IL-6 stimulation significantly increased sEV yield (Fig. 1E and F). The increased sEV yield likely results from two synergistic effects. First, IL-6 can promote MSC proliferation [36], leading to a larger cell population and thus higher total EVs production. Second, certain stimuli, such as nanoparticles, has been demonstrated to induce unconventional exocytosis (via MVBs and lysosomes) in mammalian cells, accompanied by enhanced secretion of sub-100 nm, CD81-enriched exosomes [37]. More importantly, IL-6 stimulation conferred superior biological activities compared to naïve sEVs. In LPS-stimulated RAW264.7 macrophages, E-sEVs more potently suppressed pro-inflammatory cytokines (TNF-α, iNOS) while elevating anti-inflammatory mediators (IL-10, Arg-1). Flow cytometry confirmed that E-sEVs more effectively drove M0 and M1 macrophage polarization toward the reparative M2 phenotype. These findings align with a growing body of literature demonstrating that preconditioning strategies, such as IFN-γ, TNF-α, or hypoxic exposure, significantly enhance the immunomodulatory capacity of MSC-derived sEVs [[22], [23], [24]]. Palamà and colleagues reported that hypoxic preconditioning in serum-free cultures enriched MSC-sEVs with chondroprotective miRNAs and augmented anti-inflammatory efficacy [38]. Our study extends this paradigm by establishing, for the first time, the application of IL-6 preconditioning for CEI therapy, demonstrating its superiority in modulating the local inflammatory microenvironment. Beyond immunomodulation, E-sEVs exhibited enhanced antioxidant capacity, more effectively scavenging ROS and preserving mitochondrial membrane potential in H_2_O_2_-stressed endothelial and epithelial cells. Furthermore, in TGF-β-induced fibroblast fibrosis models, E-sEVs demonstrated superior inhibition of Col I and α-SMA expression compared to naïve sEVs. Collectively, these results establish IL-6 preconditioning as an effective strategy to potentiate MSC-sEVs, likely through enrichment of a therapeutic cargo that underpins their pleiotropic actions in the complex CEI pathological milieu.

On the other hand, the esophagus presents unique challenges for local drug delivery. Its hollow, muscular structure generates continuous peristaltic activity and luminal flow that rapidly clear conventional formulations from the target site. Thus, developing a delivery system with robust mucoadhesion and sustained release capability is essential for effective CEI therapy. Cationic hyaluronic acid (CHA), a hyaluronic acid derivative modified with positively charged groups, offers an ideal platform, as its cationic nature enables electrostatic interaction with negatively charged cell surfaces and extracellular matrix components, thereby prolonging therapeutic retention [31,32]. We systematically characterized CHA formulations across concentrations (5-20% w/v). Integrating SEM microstructural analysis, adhesion testing on multiple surfaces (including porcine skin to simulate esophageal tissue), and simulated intraesophageal instillation, 15% (w/v) was identified as the optimal concentration. This formulation achieves sufficient adhesive force to resist esophageal peristalsis while maintaining injectability through gavage needles, enabling minimally invasive administration. In vitro release kinetics demonstrated rapid E-sEV liberation, with over 70% released within 6 h, ensuring prompt bioavailability at the injured site. In the animal study, the fluorescently labeled E-sEVs remained localized at the esophageal injury site for less than 3 h, after which the signal gradually diminished. This relatively short retention time represents a potential limitation of the current delivery system. However, we reasoned that this brief exposure may still be sufficient to initiate therapeutic effects, as sEVs are rapidly internalized by target cells and can trigger sustained downstream signaling cascades even after the vesicles have been cleared from the extracellular space. In addition, the initial burst of local EV retention from CHA hydrogel likely enhanced the bioavailability of sEVs during the critical early phase of wound healing. Nevertheless, it was acknowledged that repeated administration or chemical modification of the hydrogel could further prolong EVs retention and potentially improve long-term outcomes. This issue warrants systematic investigation in future studies. Notably, when sEVs or E-sEVs were loaded into CHA, their pro-migratory and pro-angiogenic effects were significantly enhanced compared to individual components or simple mixtures. This synergistic amplification indicates that CHA functions not as an inert carrier but as an active contributor to therapeutic efficacy. Particularly striking was CHA's intrinsic capacity to induce endothelial tube formation, consistent with hyaluronic acid's native role as an extracellular matrix constituent promoting angiogenesis [39]. The addition of E-sEVs further amplified this effect, exemplifying the carrier-cargo synergy that constitutes the core design advantage of the E-sEVs@CHA composite system. What's more, CHA showed excellent biocompatibility and biosafety. The absence of cytotoxicity for our CHA formulation can be attributed to two key factors. First, the degree of cationization is only 23%, resulting in a low surface charge density that is insufficient to cause significant membrane disruption. Second, CHA forms a gel-like polymeric structure rather than discrete nanoscale particles, which further reduces the risk of intensive membrane interactions and pore formation. This stands in marked contrast to highly cationic lipids such as Lipofectamine 2000 and PEI, which exist as small nanoparticles with high charge density and are known to induce pronounced cytotoxicity and immune responses at comparable concentrations [40].

The pathophysiology of CEI progresses through three overlapping phases: acute necrosis, inflammatory response, and chronic fibrosis/stricture formation [41]. An ideal therapeutic strategy must address this temporal cascade through multi-phasic, multi-target interventions. In the early stage of NaOH-induced injury, overwhelming oxidative stress and inflammatory cascades drive tissue destruction and initiate subsequent fibrogenesis. Our results demonstrate that E-sEVs@CHA intervention at day 8 post-injury significantly reduced esophageal tissue mRNA expression of pro-inflammatory cytokines (IL-1β, IL-6, TNF-α) while elevating anti-inflammatory IL-10. These in vivo observations corroborate our in vitro macrophage polarization findings, confirming that E-sEVs@CHA effectively shifts the inflammatory microenvironment from M1-dominant to M2-reparative phenotypes. Concomitantly, E-sEVs@CHA markedly reduced H_2_O_2_-induced ROS accumulation in endothelial cells and restored depolarized mitochondrial membrane potential. It is proposed that this seemingly paradoxical regulation reflects sophisticated redox homeostasis re-establishment. Supporting this interpretation, recent studies have demonstrated that umbilical cord MSC-derived exosomes attenuate oxidative stress and inflammation through modulation of the NRF2/NF-κB/NLRP3 signaling pathway [42]. Furthermore, MSC-derived exosomes have been shown to regulate the Keap1/Nrf2 axis in degenerative disease models, restoring antioxidant defense systems [43].

At the chronic phase, fibrosis represents the ultimate determinant of poor prognosis in CEI patients. At day 28 post-injury, E-sEVs@CHA-treated animals exhibited the mildest esophageal stricture severity, smallest collagen deposition area, and most significantly suppressed mRNA expression of fibrotic markers (TGF-β, α-SMA, Col I). These findings align with the seminal work by Lai and colleagues, who demonstrated that MSC-derived exosomes encapsulated in chitosan/gelatin sponges prevented esophageal stricture in a porcine endoscopic submucosal dissection model *via* MFGE8-induced M2b macrophage polarization [16]. Our study extends these observations by demonstrating that IL-6-primed E-sEVs, combined with CHA-mediated sustained delivery, achieve synergistic anti-fibrotic efficacy exceeding that of naïve sEVs. The mechanisms likely involve direct inhibition of TGF-β/Smad signaling, a pathway extensively documented in MSC-exosome anti-fibrotic effects in hepatic fibrosis models, coupled with long-term remodeling of the inflammatory microenvironment. Critically, our findings must be contextualized against earlier cell-based approaches. Kantarcioglu and colleagues reported that a single intravenous bolus of MSCs failed to improve esophageal injury in rats, despite demonstrating MSC homing and differentiation at the injury site [44]. This apparent discrepancy highlights the critical importance of both cell source (primed sEVs versus naïve MSCs) and delivery strategy (repeated local administration via bioadhesive hydrogel versus single systemic infusion) in overcoming the therapeutic ceiling observed in earlier studies. Our repeated, local E-sEVs@CHA administration likely ensures sustained therapeutic payload at the target site, addressing the limitations of transient systemic exposure.

Furthermore, through the integration of small RNA sequencing and proteomic analysis, this study preliminarily elucidates the multi-dimensional molecular regulatory network by which E-sEVs promote the repair of esophageal corrosive injury. It is found that the target genes of three significantly differentially expressed miRNAs (miR-1246, miR-193a-5p, miR-1290) in E-sEVs are enriched in key signaling pathways such as PI3K-Akt, MAPK, and HIF-1. Concurrently, the differentially expressed proteins are significantly enriched in the JAK-STAT pathway and ribosome function-related terms. This suggests that E-sEVs may remodel the injury microenvironment through the coordinated regulation of post-transcriptional modification and protein translation processes. Notably, the significant downregulation of miR-1246 in E-sEVs may be a core event underlying their reparative effects. Based on previous research, miR-1246 can directly target the 3′-UTR of PIK3AP1 and inhibit its expression, thereby negatively regulating the PI3K-Akt pathway [45]. The downregulation of miR-1246 observed in this study could theoretically relieve the inhibition on PIK3AP1, activate PI3K-Akt signaling, and consequently promote esophageal epithelial cell proliferation, anti-apoptosis, and migration. Simultaneously, the downregulation of miR-1246 may also relieve its inhibition on the MAPK/AP-1 pathway, leading to moderate activation of p-ERK, p-JNK, and c-Jun/c-Fos, restoring MMP-1 to normal levels [46]. This would facilitate epithelial remodeling and wound healing while avoiding excessive collagen degradation. Additionally, the upregulation of extracellular matrix components such as COL1A2, COL5A1, and VCAN in E-sEVs suggests they may promote cell anchorage and migration by providing a provisional matrix scaffold [47]. Conversely, the downregulation of inflammatory chemokines like IL-6, CCL2, and CXCL1 may help suppress excessive immune responses and mitigate secondary injury [48]. Notably, the downregulation of complement components (C5, C7, C9) and coagulation factors (F9, F10) also implies that E-sEVs may foster a regenerative microenvironment by modulating innate immunity and the coagulation cascade [49]. Pathway interaction analysis reveals that JAK-STAT, acting as a signaling integration hub, engages in extensive crosstalk with the PI3K-Akt, MAPK, and HIF-1 pathways [50,51]. The significant enrichment of the JAK-STAT pathway in this study, combined with the upregulation of ribosomal proteins and histone families, suggests that E-sEVs may promote rapid cell proliferation and differentiation post-injury by enhancing protein synthesis capacity and chromatin remodeling. Furthermore, the upregulation of CGAS is noteworthy, potentially implicating it in DNA damage repair and inflammatory regulation via the STING pathway [52], although the specific mechanisms require further validation.

## 5 Conclusions

In this study, we successfully developed a composite hydrogel system, E-sEVs@CHA, by integrating IL-6-primed MSC-derived small extracellular vesicles with a bioadhesive cationic hyaluronic acid scaffold. This system demonstrated potent anti-inflammatory, antioxidant, and anti-fibrotic effects both in vitro and in a rat model of corrosive esophageal injury. By synergistically enhancing the functional properties of sEVs through cytokine preconditioning and ensuring their sustained retention via CHA-mediated adhesion, E-sEVs@CHA effectively modulated the local microenvironment, promoted tissue regeneration, and inhibited esophageal stricture formation. Our findings highlight the therapeutic potential of combining preconditioned sEVs with bioadhesive materials for the treatment of esophageal injuries, offering a promising and translatable strategy for clinical management of CEI.

## CRediT authorship contribution statement

**Dan Luo:** Writing – original draft, Project administration, Funding acquisition, Formal analysis, Data curation. **Shixian Li:** Writing – original draft, Investigation, Formal analysis, Data curation. **Jianhong Sun:** Methodology, Investigation. **Shaolong Zheng:** Resources, Investigation. **Zhengkui Huang:** Visualization, Methodology. **Na Xiang:** Formal analysis, Data curation. **Shan Guo:** Software, Resources. **Zhi Yu:** Visualization, Software. **Hong Mei:** Visualization, Resources. **Na Xu:** Writing – review & editing, Supervision, Resources. **Xiaohui Song:** Supervision, Resources, Project administration. **Baoxiang Wang:** Writing – review & editing, Supervision, Conceptualization.

## Funding declaration

This work was financially supported by the 10.13039/501100003819Natural Science Foundation of Hubei Province (No. 2022CFB442), the Funding for Scientific Research Projects from Wuhan Municipal Health Commission (No. WX21C06), and the Science Research Foundation of Department of Education of Hubei Province of China (No. Q20201110).

## Declaration of competing interest

The authors declare that the research was conducted in the absence of any commercial or financial relationships that could be construed as a potential conflict of interest.

## Data Availability

Data will be made available on request.
