# Supplementary material for: Adhesive cationic hyaluronic acid loading with small extracellular vesicles from IL-6 activated mesenchymal stem cells improves repair of corrosive esophageal injury
Source: Mater Today Bio. 2026 Sep 23;41:103706. doi: 10.1016/j.mtbio.2026.103706 (PMC13656951; doi:10.1016/j.mtbio.2026.103706)
Supplement: Supplementary file 1 [file mmc1.docx]

**Supplementary Material**

**Figure S1** The chemical structure of CHA.

**Figure S2** (A) Viscosity of CHA at different concentrations. (B) Injectability macroscopic image of 15% CHA.

**Figure S3** Biocompatibility evaluation of E-sEVs@CHA. (A) Live/dead cell staining images (scale bars: 200 μm). (B) CCK-8 detection results (n=4). (C) Macroscopic images of hemolysis test. (D) Quantitative analysis of hemolysis test (n=3). (ns means no significance, * p < 0.05, ** p < 0.01, *** p < 0.001）

**Figure S4** Biosafety of CHA. (A) H&E-stained sections of esophagus, heart, liver, spleen, lung, kidney after intraesophageal instillation with CHA on day 28. (B) Blood biochemical indicators detection of alanine aminotransferase (ALT), aspartate aminotransferase (AST), AST/ALT ratio, creatinine (CR), uric acid (UA), and urea nitrogen (BUN). (ns, P > 0.05, n=3)

**Figure S5** Construction of the rat CEI model induced by NaOH. (A) H&E-stained sections of esophagus after injury with different concentrations (w/v) of NaOH on days 7, 14, and 21. (B) Masson-stained sections of esophagus after injury with different concentrations (w/v) of NaOH on day 28. (C) Esophagography images after injury with different concentrations (w/v) of NaOH on day 28.

**Figure S6** In vivo retention of sEVs@CHA and E‑sEVs@CHA at the esophagus at different time points imaged by IVIS^®^ Spectrum Imaging System.
